# A combination of potently neutralizing monoclonal antibodies isolated from an Indian convalescent donor protects against the SARS-CoV-2 Delta variant

**DOI:** 10.1371/journal.ppat.1010465

**Published:** 2022-04-28

**Authors:** Nitin Hingankar, Suprit Deshpande, Payel Das, Zaigham Abbas Rizvi, Constantinos Kurt Wibmer, Poppy Mashilo, Mohammed Yousuf Ansari, Alison Burns, Shawn Barman, Fangzhu Zhao, Sohini Mukherjee, Jonathan L. Torres, Souvick Chattopadhyay, Farha Mehdi, Jyoti Sutar, Deepak Kumar Rathore, Kamal Pargai, Janmejay Singh, Sudipta Sonar, Kamini Jakhar, Jyotsna Dandotiya, Sankar Bhattacharyya, Shailendra Mani, Sweety Samal, Savita Singh, Pallavi Kshetrapal, Ramachandran Thiruvengadam, Gaurav Batra, Guruprasad Medigeshi, Andrew B. Ward, Shinjini Bhatnagar, Amit Awasthi, Devin Sok, Jayanta Bhattacharya

**Affiliations:** 1 IAVI HIV Vaccine Translational Research Laboratory, IAVI-THSTI partnership program, Translational Health Science & Technology Institute, NCR Biotech Science Cluster, Faridabad, India; 2 Immuno-biology Lab, Translational Health Science & Technology Institute, NCR Biotech Science Cluster, Faridabad, India; 3 Immunology Core, Translational Health Science & Technology Institute, NCR Biotech Science Cluster, Faridabad, India; 4 National Institute for Communicable Diseases (NICD) of the National Health Laboratory Service (NHLS), Johannesburg, South Africa; 5 Scripps Consortium for HIV/AIDS Vaccine Development (CHAVD), The Scripps Research Institute, La Jolla, California, United States of America; 6 IAVI Neutralizing Antibody Center and the Collaboration for AIDS Vaccine Discovery (CAVD), The Scripps Research Institute, La Jolla, California, United States of America; 7 Department of Immunology and Microbiology, The Scripps Research Institute, La Jolla, California, United States of America; 8 IAVI, New York, United States of America; 9 IAVI, New Delhi, India; 10 Department of Integrative Structural and Computational Biology, The Scripps Research Institute, La Jolla, California, United States of America; 11 Translational Health Science & Technology Institute, NCR Biotech Science Cluster, Faridabad, India; 12 Bioassay laboratory, Translational Health Science & Technology Institute, NCR Biotech Science Cluster, Faridabad, India; Institut Pasteur, FRANCE

## Abstract

Although efficacious vaccines have significantly reduced the morbidity and mortality of COVID-19, there remains an unmet medical need for treatment options, which monoclonal antibodies (mAbs) can potentially fill. This unmet need is exacerbated by the emergence and spread of SARS-CoV-2 variants of concern (VOCs) that have shown some resistance to vaccine responses. Here we report the isolation of five neutralizing mAbs from an Indian convalescent donor, out of which two (THSC20.HVTR04 and THSC20.HVTR26) showed potent neutralization of SARS-CoV-2 VOCs at picomolar concentrations, including the Delta variant (B.1.617.2). One of these (THSC20.HVTR26) also retained activity against the Omicron variant. These two mAbs target non-overlapping epitopes on the receptor-binding domain (RBD) of the spike protein and prevent virus attachment to its host receptor, human angiotensin converting enzyme-2 (hACE2). Furthermore, the mAb cocktail demonstrated protection against the Delta variant at low antibody doses when passively administered in the K18 hACE2 transgenic mice model, highlighting their potential as a cocktail for prophylactic and therapeutic applications. Developing the capacity to rapidly discover and develop mAbs effective against highly transmissible pathogens like coronaviruses at a local level, especially in a low- and middle-income country (LMIC) such as India, will enable prompt responses to future pandemics as an important component of global pandemic preparedness.

## Author summary

SARS-CoV-2, a highly infectious and pathogenic virus that caused the COVID-19 pandemic, has led to unprecedented devastation and disruption in human health, public safety, and the global economy. The virus genome sequence has aided in the rapid development of vaccines as well as the discovery of potential therapeutic agents such as monoclonal antibodies (mAbs). In India, the emergence of the Delta variant led to a significant rise in caseloads and deaths that overwhelmed health systems. Here, we describe the isolation of novel cross SARS-CoV-2 variant neutralizing monoclonal antibodies (mAbs) from a single Indian convalescent individual, two of which (THSC20.HVTR04 and THSC20.HVTR26) demonstrated very potent neutralization of SARS-CoV-2 variants of concern (VOC) by targeting non-overlapping epitopes in the spike protein’s receptor binding domain (RBD). One of these two mAbs, THSC20.HVTR26 also retained activity against the Omicron variant albeit with reduced potency. Furthermore, in a hACE2 transgenic mouse model, passive transfer of their combination conferred significant protection against infection by Delta variant at a low dose, implying that these two highly potent mAbs have the potential to be developed as products for therapeutic and preventive application against SARS-CoV-2 infection. The lessons learned from the COVID-19 pandemic emphasize the importance of a timely response through the discovery of effective mAbs as part of pandemic preparedness for future emerging infectious diseases.

## Introduction

SARS-CoV-2 is a positive-sense single stranded RNA virus that is the etiological cause of COVID-19, which has led to over 5 million deaths globally (https://covid19.who.int/). Although SARS-CoV-2 has a relatively low mutation rate, a combination of high transmission events (over 250 million cases to date), inequitable vaccine access, and prevailing vaccine hesitancy, has led to the selection and spread of variants of concern (VOCs) that drive this persisting pandemic. These VOCs (https://www.who.int/en/activities/tracking-SARS-CoV-2-variants/) have garnered mutations that give them a selective advantage, either higher transmission, resistance to vaccine responses, or both. Among all of the SARS-CoV-2 VOCs, the Delta variant (B.1.617.2), first detected in India in late 2020 in the state of Maharashtra [1,2], became the globally dominant circulating strain during the period from July 2021 to December 2021 (https://nextstrain.org/ncov/gisaid/global). The Delta variant led to a large spike in COVID-19 cases in India as part of a second wave that culminated in over 30 million cases and over 400,000 deaths [1,3]. Even though the third wave in India was dominated by the Omicron variant (over 60% of total sequences analyzed in February 2022), analysis of virus sequences reported from India in the GISAID database (https://www.epicov.org/) indicated that Delta variant (over 20% of the total sequences analyzed) continued to infect till February 2022. Since the first case of Delta was reported, scientists have determined that the Delta variant is more than twice as transmissible as the original strain of SARS-CoV-2 [2]. Some studies have also indicated that infection with the Delta variant leads to higher viral loads and worse disease prognosis compared to the original Wuhan strain [4]. Importantly, countries with high vaccination rates observed increases in cases due to Delta, indicating some breakthrough infection, but did not observe a proportional increase in hospitalization [5,6]. Unfortunately, countries with limited access to vaccines face higher rates of COVID-19 cases and increases in hospitalization, which could overwhelm health systems and add to the growing tally of morbidity and mortality due to COVID-19.

Since the emergence of Delta, a new SARS-CoV-2 variant called Omicron has captured the attention of scientists and public health officials. Omicron contains a much larger number of mutations than Delta when compared to the original Wuhan strain. Recent studies have indicated that Omicron is more transmissible than the Delta variant [7–10] and vaccine responses are less effective against Omicron [11,12]. As access to and uptake of efficacious vaccines remain inequitable across the globe, the likelihood of additional VOCs emerging is high, particularly among immunocompromised individuals—notably people living with HIV (www.hiv.gov) who are not able to reliably access treatment and the millions of people around the world who are receiving treatment for cancer and other related diseases. This combination of factors leads to a perpetual unmet medical need for treatment options as global vaccine distribution and uptake languish.

In the absence of efficacious small molecule drugs at the height of the pandemic, monoclonal antibodies (mAbs) were discovered and developed as a relatively rapid countermeasure to prevent hospitalization. While effective drugs can take up to a decade or longer to discover and develop, Eli Lilly and Regeneron [13] were able to advance mAbs from discovery to emergency use authorization (EUA) in an unprecedented 8 months. These mAbs have variable activity against different VOCs, including loss of neutralization activity of Bamlanivimab against Delta and Beta variants, loss of activity of Etesivimab against Beta and reduced activity against Alpha variant, and reduced activity of Casirivimab against the Beta variant. Imdevimab, which is delivered in combination with Casirivimab as part of the REGN-CoV cocktail, remains active against Alpha, Beta, and Delta variants. The new Omicron variant, however, appears to be resistant to all four EUA antibodies [14–16]. To address resistant VOCs like Omicron and others that might arise, other programs have focused [14] on isolating neutralizing antibodies that target conserved epitopes on the spike protein and are therefore hypothesized to remain active against current and future VOCs. A complementary approach is to establish capacity in low- and middle-income countries (LMICs) to isolate monoclonal antibodies from convalescent donors in regions where VOCs are likely to emerge. Studies have shown that despite reduced neutralization activity against VOCs of sera from vaccinated individuals or convalescent donors infected with the original strain, those who are infected with VOCs like Beta do mount a potent neutralizing antibody response [17]. These findings highlight the value of isolating new mAbs from convalescent donors that are effective against new VOCs to provide additional therapeutic options to prevent severe COVID-19 disease.

In the present study, we report isolation of monoclonal antibodies (mAbs) from an Indian convalescent donor by antigen-specific single B cell sorting using the receptor binding domain (RBD) of SARS-CoV-2 Wuhan strain. We report the discovery of two monoclonal antibodies (THSC20.HVTR04 and THSC20.HVTR26) that have non-competing epitope specificities on the RBD and show potent neutralization of Alpha, Beta, Gamma, and Delta VOCs. Furthermore, a combination of these two mAbs demonstrated significant *in vivo* protection at low antibody dose against Delta challenge in K18 hACE2 transgenic mice.

## Results

### Identification of a convalescent donor with high serum antibody titers and neutralizing activity against SARS-CoV-2 VOCs

**Fig 1:**
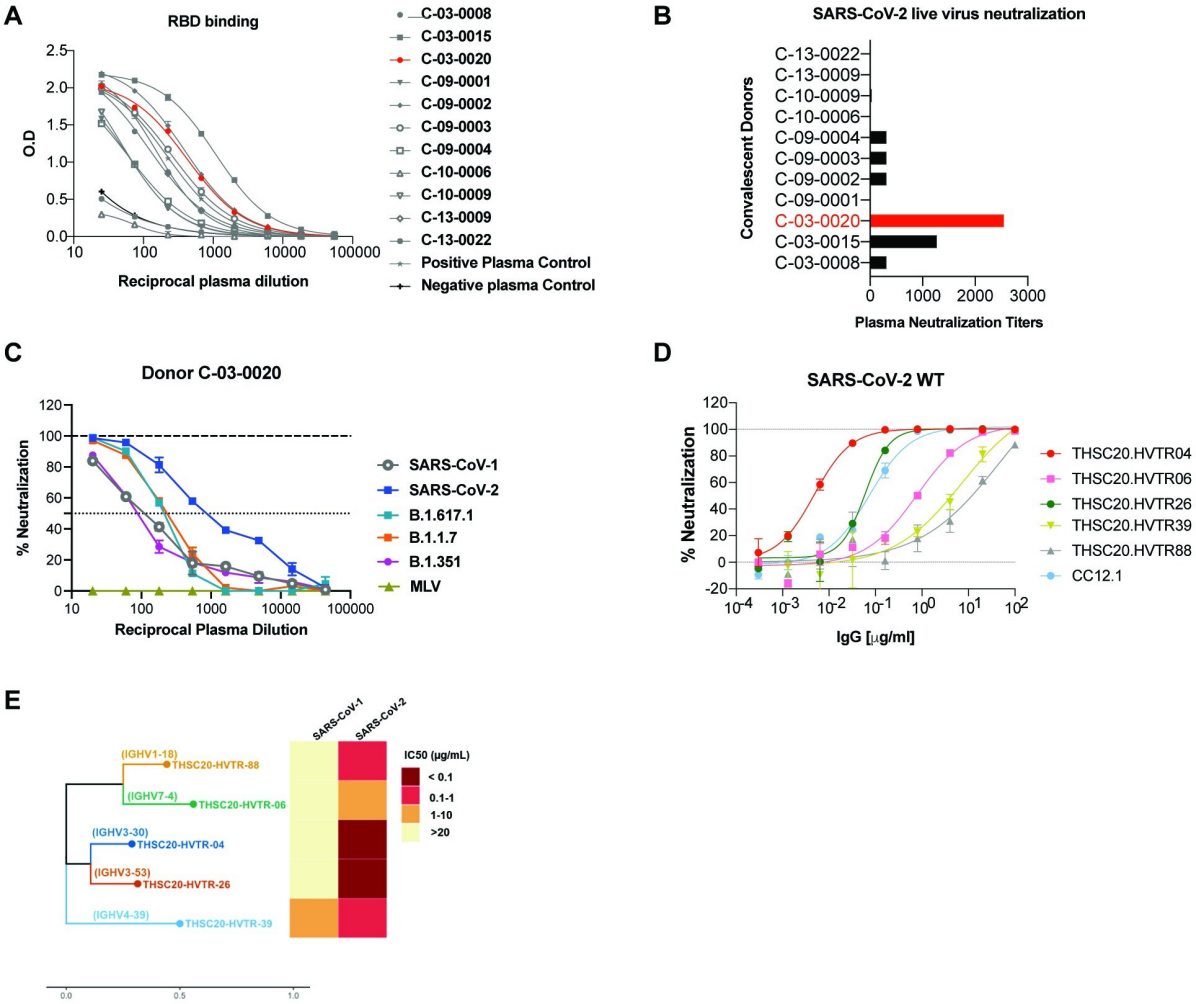
Isolation of neutralizing antibodies from the memory B cells of a convalescent donor. **A**. Identification of C-03-0020 donor with significant binding to SARS-CoV-2 RBD. **B**. High neutralizing activity of C-03-0020 plasma against SARS-CoV-2 in live virus neutralization assay. **C**. High neutralizing activity of C-03-0020 plasma against SARS-CoV-2 in pseudovirus neutralization assay. **D.** Neutralization potential of RBD-reactive mAbs were evaluated against SARS-CoV-2 pseudovirus. **E**. CDRH3 sequence, germline usage neutralization potency of isolated mAbs against SARS-CoV and SARS-CoV-2.

We first screened the plasma samples obtained 6–8 weeks post infection from eleven convalescent donors in the DBT India COVID-19 Consortium cohort [18] who recovered from COVID-19 (S1 Table). These samples were screened for anti-RBD serum titers and neutralizing activity against SARS-CoV-2 VOCs using Vero-E6 as target cells. Through this evaluation, we identified one convalescent donor (C-03-0020) whose plasma exhibited strong binding to RBD by ELISA **(Fig 1A)**, neutralization of live SARS-CoV-2 virus (Wuhan strain) (**Fig 1B**), and broad neutralization of SARS-CoV-2 (Wuhan isolate), SARS-CoV-2 VOCs, and SARS-CoV pseudoviruses **(Fig 1C)**. Based on these data, we next attempted to isolate RBD-specific monoclonal antibodies from donor C-03-0020.

### Isolation of RBD-specific anti-SARS-CoV-2 monoclonal antibodies (mAbs)

Although anti-SARS-CoV-2 antibodies targeting different regions of the viral spike such as RBD, S1, S2 domains and N-terminal domain (NTD) have been reported [13,19–29], most of the potently neutralizing mAbs target the RBD of SARS-CoV-2, presumably because the mAbs compete with ACE2 binding. We therefore utilized RBD as antigen bait to rapidly isolate anti-SARS-CoV-2 mAbs using single B cell FACS sorting method as described previously [20]. Using this strategy, a total of 48 SARS-CoV-2 RBD specific single B cells were antigen-sorted **(S1 Fig),** and mRNA from sorted cells were reverse transcribed and heavy and light chain variable regions were subsequently amplified by PCR. We successfully amplified heavy and light chains from 38 out of 48 RBD-specific B cells (79% efficiency), which were then cloned into expression vectors and produced as recombinant monoclonal antibodies. Antibody transfection supernatants from these 38 heavy and light chain pairs were next screened for expression and binding to SARS-CoV-2 RBD by ELISA. As shown in **S2 Fig,** 5/ 38 mAb supernatants showed expression by Fc capture ELISA and binding to SARS-CoV-2 RBD **(Fig 1D)**. While all the five mAbs as purified IgGs neutralized pseudovirus expressing spikes of SARS-CoV-2 wild type, Alpha, Beta and Kappa (with IC50 ranging from 0.003–7.2 μg/mL), one of the mAbs (THSC20.HVTR39) showed evidence of neutralizing both SARS-CoV and SARS-CoV2. (**Figs 1 and S4)**.

Sequence analysis revealed that all the five neutralizing mAbs derived from distinct germline lineages as shown in **Fig 1E and S2 Table**, indicating a polyclonal neutralizing antibody response following infection in this donor. Except for THSC20.HVTR88, which has a kappa light chain, all the other four mAbs (THSC20.HVTR04, THSC20.HVTR06, THSC20.HVTR26 and THSC20.HVTR39) have lambda light chains. We also note that mAb THSC20.HVTR26 is derived from the IGHV3-53 heavy chain gene, which was reported to be a common variable heavy chain gene for SARS-CoV-2 nAbs **(S2 Table)** [30]. We next evaluated the level of somatic hypermutation in the variable genes. Similar to previously reported findings [20,28,31,32], the isolated nAbs have relatively low levels of somatic mutations that range between 94.5% to 98.25 identical to germline. The H-CDR3 length of isolated mAbs range from 16 to 23 amino acids (aa). For the corresponding light chains, the level of IGLV gene somatic mutation ranges from 93.01 to 97.92, while the mAb with a kappa chain is 100% identical to its IGKV germline **(S2 Table).**

### Neutralizing antibodies are active against SARS-CoV-2 VOCs

**Fig 2:**
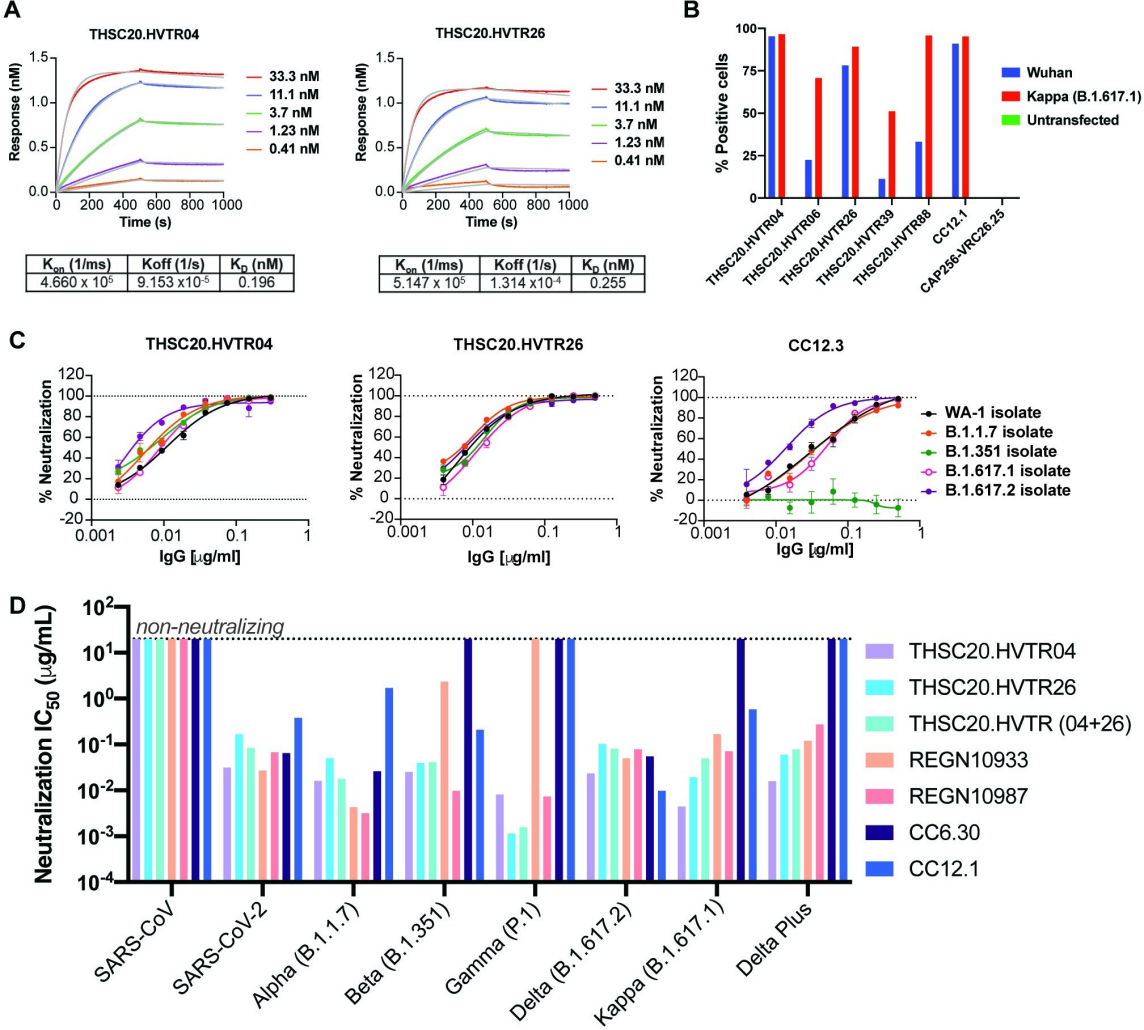
Isolated monoclonal antibodies can neutralize all circulating variants of concern and interest. **A**. Binding affinities of THSC20.HVTR04 and THSC20.HVTR26 to the SARS-CoV-2 (Wuhan) receptor binding domain (RBD) protein by BLI-Octet. Biotinylated wild type SARS-CoV-2 RBD antigen was immobilized on Streptavidin (SA) biosensors and binding affinity of monoclonal antibodies to RBD was tested using three-fold serial dilutions of mAbs starting with 33.3 nM and lowest 0.41 nM (five different concentrations were tested). Association and dissociation was assessed for 500 seconds each. Data shown is reference-subtracted and aligned using Octet Data Analysis software v11.1 (Forte Bio). Curve fitting was performed using a 1:1 binding model and K_on_, K_off_ and K_D_ values were determined with a global fit. **B**. Binding of mAbs to SARS-CoV-2 spike protein expressed on 293T cells as assessed by mean fluorescent intensity (MFI) in a flow cytometry. **C**. Live virus focus reduction neutralization assay. The ability of the two top mAbs (THSC20.HVTR04 and THSC20.HVTR26) was assessed by dose-dependent foci reduction neutralization (FRNT) live virus neutralization assay in Vero-E6 cells. **D**. Expanded pseudovirus neutralization assay of THSC20.HVTR04 and THSC20.HVTR26 against circulating VOCs (Alpha, Beta, Gamma, Delta) and VOIs (Kappa, Delta Plus). Other known mAbs (REGN10933, REGN10987, CC12.1 and CC6.36) were included in the experiment as benchmarking controls. Representative dose response curves are shown with each concentration response tested in duplicate. Values shown are mean with SEM.

Next, we examined the ability of the newly isolated mAbs to neutralize the SARS-CoV-2 VOCs (Alpha, Beta, Gamma, and Delta) and variants of interest (VOIs) that have circulated in India (Kappa, B.1.36 and Delta plus). First, we examined the binding affinity and avidity of the five newly isolated mAbs to the SARS-CoV-2 RBD by biolayer interferometry (BLI) and ELISA respectively. As shown in **Figs 2A and S3A**, BLI analysis of these five mAbs showed apparent RBD binding affinity between 0.19–0.813 nM, with THSC20.HVTR04 having the highest apparent affinity (0.19 nM) and THSC20.HVTR39 having the lowest apparent affinity (0.813 nM). These values closely match apparent affinity measurements by ELISA to RBD with EC50 values ranging from 0.19–0.06 μg/mL **(S3B Fig)**. THSC20.HVTR04 and THSC20.HVTR26 showed the highest affinity in these assays.

Since we used monomeric RBD to isolate the mAbs, we further assessed their ability bind to trimeric spike protein expressed on the cell surface. For this purpose, we expressed the SARS-CoV-2 spikes representing Wuhan wild-type or Kappa (B.1.617.1) variant sequences on the surface of 293T cells and measured binding of mAbs to cell surface spike by fluorescence-activated cell sorting (FACS). As shown in **Fig 2B**, THSC20.HVTR04 and THSC20.HVTR26 bound more strongly than the other mAbs to the spike proteins expressed on the surface of 293T cells.

We next evaluated the neutralization activity of the mAbs against SARS-CoV-2 variants in a pseudovirus assay. The panel of SARS-CoV-2 variants included the Wuhan strain, Alpha, Beta, and Kappa strains. We found that THSC20.HVTR04 and THSC20.HVTR26 neutralized these SARS-CoV-2 variants with the highest potency (**S4 Fig**). This observation is consistent with their high binding affinity to the RBD as shown above. Additionally, we observed that mAb THSC20.HVTR39 neutralized SARS-CoV with IC50 of 2.9 μg/mL, indicating that this particular mAb is capable of neutralizing both SARS-CoV and SARS-CoV-2.

THSC20.HVTR04 and THSC20.HVTR26 showed the greatest potency out of all the isolated mAbs. Therefore, these mAb were subsequently evaluated for neutralization both individually and in combination in a live virus focus reduction neutralization assay against the following VOC isolates: Alpha, Beta, Delta and VOI Kappa (as shown in **Figs 2C and S5**). We also compared these antibodies to previously reported neutralizing mAbs [13,20] in a pseudovirus neutralization assay (**Fig 2D**). As shown in **Fig 2C and S3 Table**, both THSC20.HVTR04 and THSC20.HVTR26 mAbs showed very potent neutralization against all VOCs and Kappa variant with IC50s ranging from 0.003–0.01 μg/mL when assessed against live virus isolates. Comparable neutralization potencies of THSC20.HVTR04 and THSC20.HVTR26 mAbs individually and in combination were also observed when assessed in a pseudovirus neutralization assay (**Fig 2D and S4 Table**). Both THSC20.HVTR04 and THSC20.HVTR26 mAbs potently neutralize Gamma (P1) and Delta-plus variants in the pseudovirus neutralization assay, which are resistant to some of the previously reported neutralizing mAbs. We note the modest superiority of these two novel mAbs over REGN10933 and REGN10987 mAbs [13] when compared head to head in the same pseudovirus neutralization assay.

**Fig 3:**
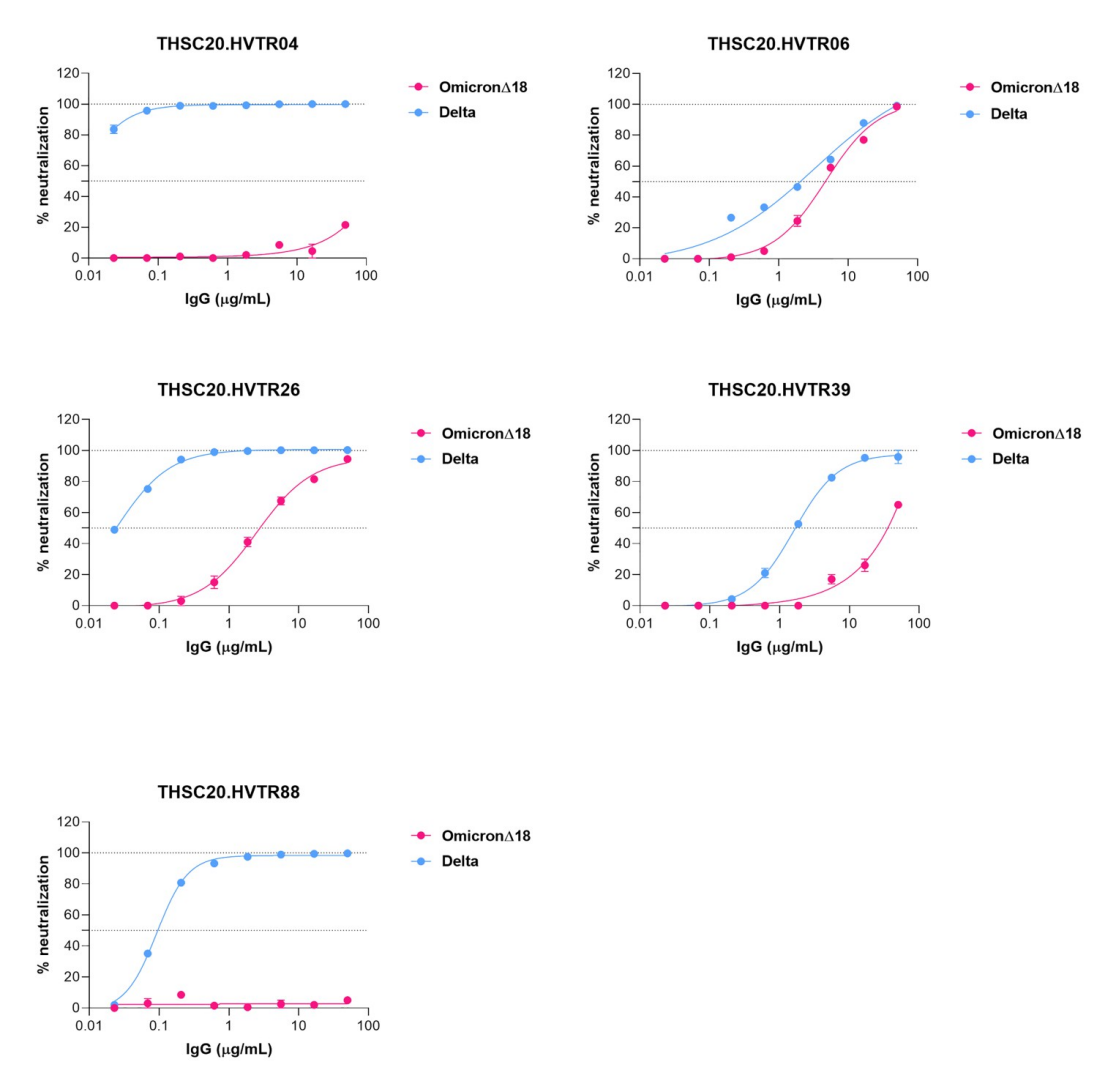
Neutralization potential of isolated monoclonal antibodies against Omicron. Neutralization of pseudovirus expressing Omicron spike by all the five newly isolated mAbs was carried out as described above. Average of three independent experiments with each concentration response tested in duplicate were used to prepare the dose response curves. Values shown are mean with SEM.

Interestingly, when assessed against Omicron variant, we found amongst all the newly isolated mAbs, THSC20.HVTR06 and THSC20.HVTR26 demonstrated neutralization of Omicron, although with reduced potencies (IC50 values of 4.14 and 2.71 μg/mL respectively), while the other mAbs were not found to demonstrate any meaningful activity against Omicron (**Fig 3**). Taken together, our data report the discovery of two highly potent mAbs (THSC20.HVTR04 and THSC20.HVTR26) from a single convalescent donor that are capable of potently neutralizing all the VOCs and VOIs tested in this study, of which two (THSC20.HVTR06 and THSC20.HVTR26) were found to neutralize Omicron variant.

### Neutralizing antibodies from the same donor target non-competing epitopes on RBD

**Fig 4:**
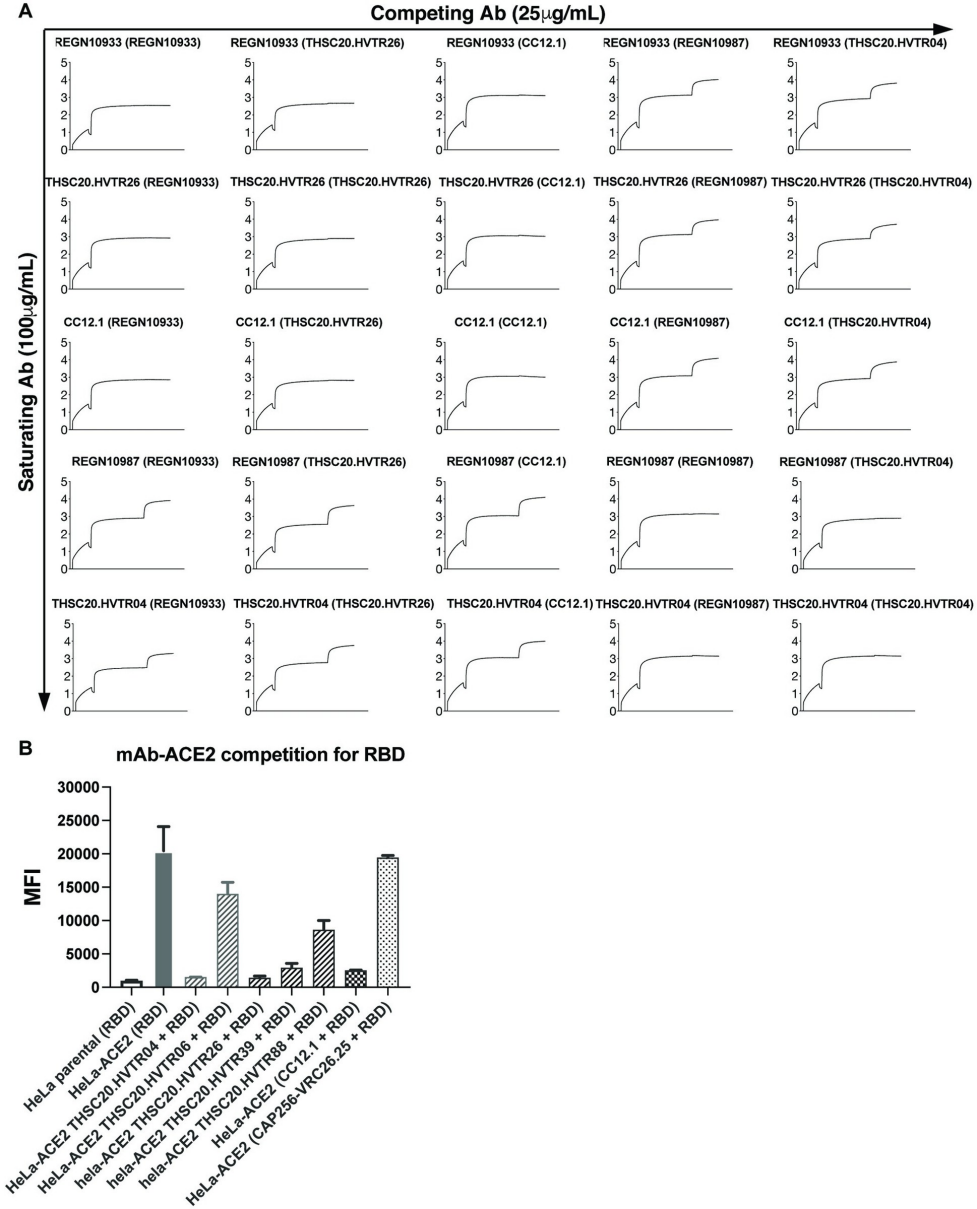
Neutralizing antibodies from the same donor target non-competing epitopes on RBD. **A.** Monoclonal antibodies were evaluated for epitope competition using BLI. Biotinylated RBD was captured using streptavidin biosensor and indicated mAbs at a concentration of 100μg/ml first incubated for 10 min followed by incubation with 25μg/ml of competing antibodies for 5 min. **B.** ACE2-mAb competition for RBD. Inhibition of SARS-CoV-2 RBD binding by five mAbs to the cell surface hACE2 was assessed by flow cytometry.

To assess the epitope specificities of the newly isolated mAbs on SARS-CoV-2 RBD, we performed epitope binning using biolayer interferometry as described previously [20]. To achieve this, biotinylated RBD was first loaded on the streptavidin biosensor followed by binding of saturating mAb at a high concentration (100 μg/mL). Subsequently, binding of a competing mAb at lower concentration (25 μg/mL) to RBD was evaluated in the presence of saturating mAb. We also tested four previously reported anti-SARS-CoV-2 antibodies (CC12.1, CC12.3, CC12.18 CC6.33.1, REGN10933 and REGN10987) [20] that target two distinct epitopes on RBD to inform our epitope binning. Our data revealed two distinct non-overlapping epitopes for THSC20.HVTR04 and THSC20-HVTR26 on the RBD. THSC20.HVTR06 and THSC20.HVTR39 did not compete with THSC20.HVTR-04 and THSC20-HVTR-26, indicating distinct epitope specificities for these mAbs **(Figs 4A and S6)**. The epitope binning experiment also revealed that THSC20.HVTR-88 and THSC20.HVTR04 have similar or overlapping epitope specificities. Compared to previously reported antibodies which were used as benchmarking antibodies in this study, our experimental data showed similar epitope specificities between THSC20.HVTR04, REGN10987, CC6.33.1 (RBD-B) and between THSC20.HVTR26, REGN10933 CC12.1 (RBD-A) antibodies, although their neutralization breadth and potency varied.

We next examined whether the isolated monoclonal antibodies compete with RBD for ACE2 receptor binding. We carried out a mAb-RBD competition for ACE2 binding assay by first incubating mAbs with RBD in a 4:1 ratio and subsequently measured binding to HeLa-ACE2 target cells by FACS. As shown in **Fig 4B**, among all the mAbs tested, THSC20.HVTR04 and THSC20.HVTR26 effectively blocked RBD binding to ACE2. We further determined which residues in the receptor binding motif (RBM) are required for antibody neutralization [33–35] for THSC20.HVTR04 and THSC20.HVTR26. We found that mutations N439K, N440K and K444N resulted in reduced sensitivity to THSC20.HVTR04, with N439K demonstrating greater than a 160-fold reduction in neutralization when assessed in pseudovirus neutralization assay **(S5 Table**). Meanwhile, the neutralization activity of THSC20.HVTR26 was unaffected by any of these point substitutions. Our data correlates with the basis for loss in sensitivity of Omicron variant to THSC20.HVTR04.

**Fig 5:**
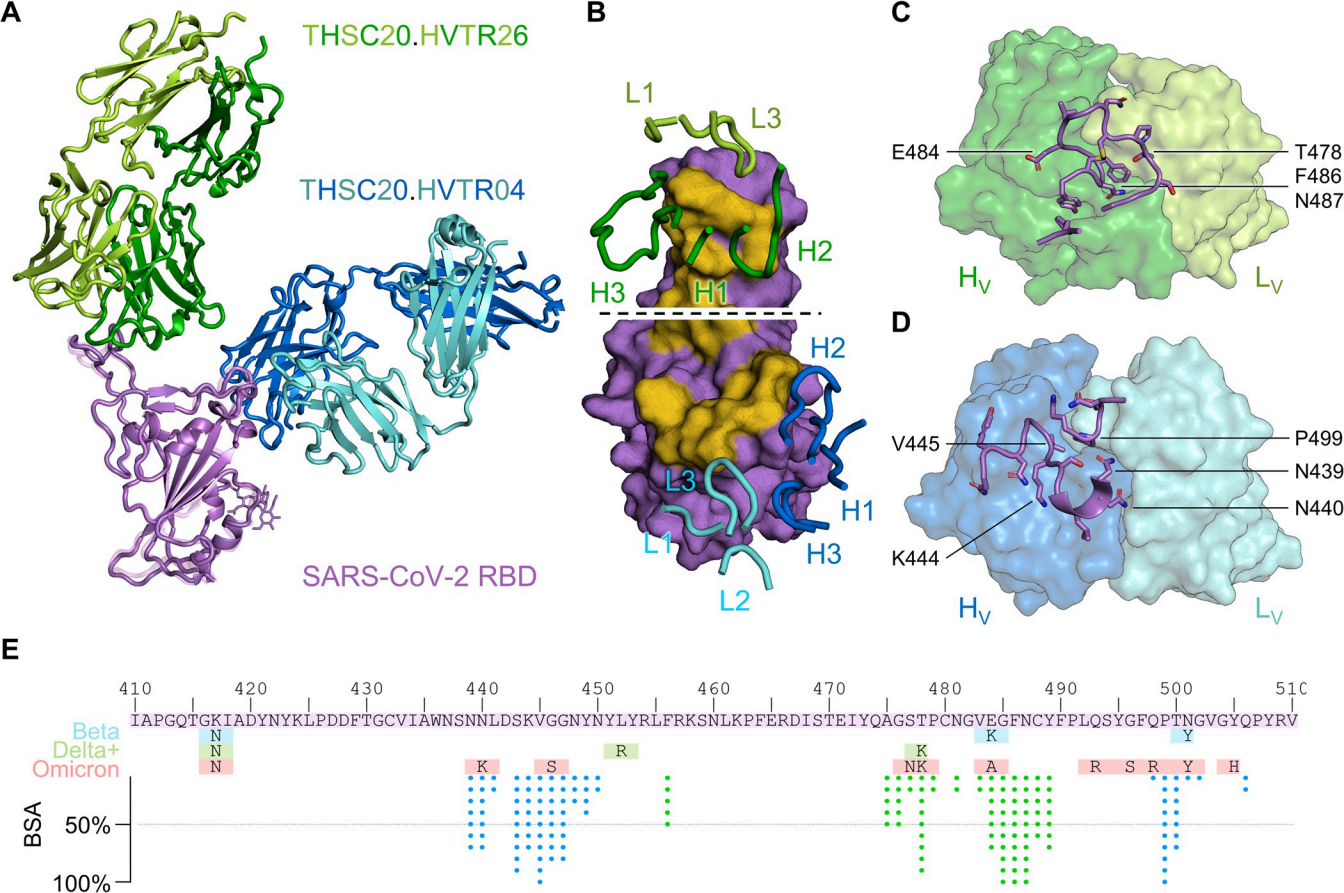
X-ray crystallography of THSC20.HVTR04 and THSC20.HVTR26 complexed with SARS-CoV-2 RBD. **A.** Schematic of THSC20.HVTR26 Fab (PDB 7Z0X, shown in dark and light green for heavy and light chains respectively) and THSC20.HVTR04 Fab (PBD 7Z0Y, shown in dark and light blue), bound to SARS-CoV-2 spike RBD (shown in purple/pink respectively). Structures were aligned by RBD. **B.** Surface view of SARS-CoV-2 RBD from PDB 7Z0X (top) or 7Z0Y (bottom), viewed from the angle of ACE2 approach, with the ACE2 binding site colored gold. Complementarity determining regions are shown in cartoon view, labelled, and colored as in A. **C.** Surface view of THSC20.HVTR26 Fab variable domains (colored as in A). RBD residues 475–489 and F456 are shown in purple cartoon and stick view. Key amino acids E484, T478, F486, and N487 are labelled. **D.** Surface view of THSC20.HVTR04 Fab variable domains (colored as in A). RBD residues 439–450 and 498–502 are shown in purple cartoon and stick view. Key amino acids V445, K444, P499, N439, and N440 are labelled. **E.** Primary sequence of SARS-CoV-2 RBD positions 410 to 510 (purple), and mutations associated with the Beta (cyan), Delta+ (lime), or Omicron (salmon) variants. The percentage of accessible surface for each amino acid that was bound by THSC20.HVTR04 or THSC20.HVTR26 was calculated (% buried surface area) and plotted using blue or green dots respectively, where each dot represents ~10% of the total solvent accessible surface area.

To further examine the structural basis for the difference in epitope specificities of THSC20.HVTR04 and THSC20.HVTR26, high resolution atomic details for THSC20.HVTR04 and THSC20.HVTR26 bound to wild type SARS-CoV-2 RBD were determined by x-ray crystallography, at 2.95 Å and 1.80 Å respectively (**Fig 5** and **S6 Table**). These data confirmed non-overlapping epitopes separated by approximately 4 Å on the same protomer, with both antibodies directly occluding the ACE2 binding site (**S7A Fig**). While THSC20.HVTR26 derived from the IGHV3-53 heavy chain gene, it had a binding mode that did not conform to the SARS-CoV-2 public antibody response ascribed to this gene. The THSC20.HVTR26 paratope was predominantly made up of residues from CDR-H2, -H3, and -L3, comprising 80% of the interface (**Figs 5B, top and S7B**). SARS-CoV-2 RBD positions G485, F486, and N487 buried all available surface area into THSC20.HVTR26, forming almost half of the epitope (**Fig 5C**). Key interactions were also made with E484 and T478. These sites are mutated in the Beta and Delta variants respectively (**Fig 5E**), both of which were neutralized by THSC20.HVTR26, suggesting that the combination of these mutations likely contributes to reduced neutralization of the Omicron variant by THSC20.HVTR26 (**S7C Fig**), although quaternary interactions may play an additional role (**S7D Fig**). The THSC20.HVTR04 paratope was more evenly distributed over all six CDRs, with heavy chain residues comprising two thirds of the interface (**Figs 5B, bottom and S7E**). Positions V445 and P499 of SARS-CoV-2 RBD were completely buried in THSC20.HVTR04, with additional key interactions at N439, N440, S443, K444 (including a salt bridge with CDR-H3 E100^C^), G446, G447, and T500 (**Fig 5D**). Of these, only N440K and G446S mutations are found in current SARS-CoV-2 VOI/VOCs (**Fig 5E**). While N440K conferred a 25-fold reduction in THSC20.HVTR04 neutralization (**see S5 Table**), the combination together with G446S and Q498R found in the Omicron variant is likely responsible for its resistant phenotype at this site (**S7E Fig**). The epitope for THSC20.HVTR04 was highly similar to Imdevimab (REGEN-COV / Ronapreve) and Bebtelovimab (**S8A Fig**), contacting the same positions in RBD (**S8C Fig**). The ability of Bebtelovimab to neutralize Omicron may be explained by subtle differences in binding angle / CDR-H2 residues that provide enough room to accommodate the G446S substitution. THSC20.HVTR26 had an epitope most closely related to Tixagevimab (Evusheld) (**S8B Fig**), and shared elements of its epitope with Bamlanivimab and Regdanvimab (**S8C Fig**), all of which display limited neutralization of, or binding to, the Omicron VOC.

**Fig 6:**
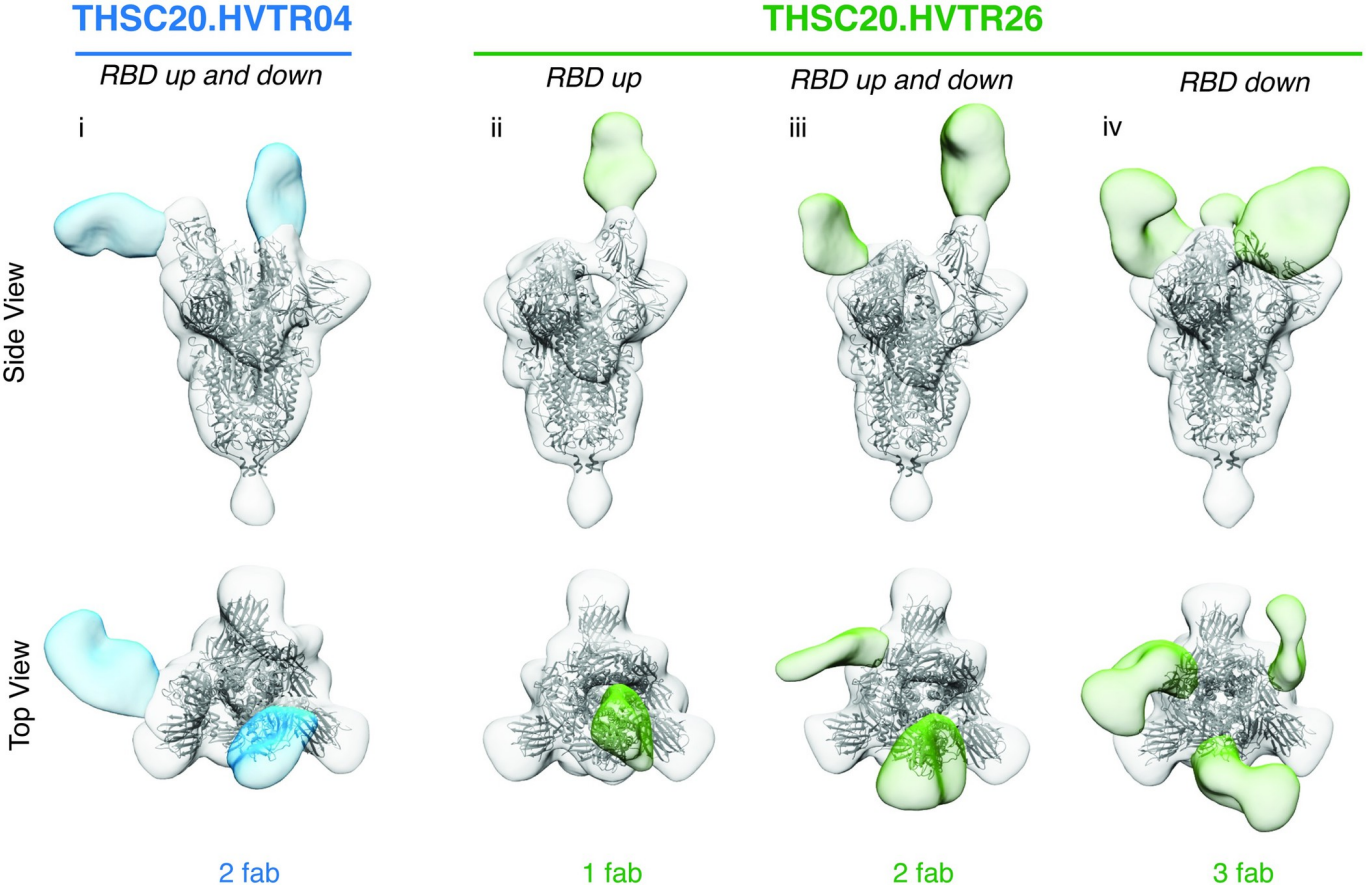
Negative stain EM analysis of mAbs complexed with SARS-CoV-2 spike protein. Negative stain EM analysis of mAbs THSC20.HVTR04 (blue) and THSC20.HVTR26 (green) complexed with SARS-CoV-2 6P Mut7 spike protein. PDB 6VYB (one RBD-up) was fit into maps i-iii, and PDB 6VXX (three RBD’s down) was fit into map iv.

Finally, we examined how THSC20.HVTR04 and THSC20.HVTR26 differ in their binding to the SARS-2 CoV 6P Mut7 spike protein ectodomain by negative stain electron microscopy (nsEM). Analysis of 2D class averages **(S9 Fig)** and 3D refinements of EM structural data indicated that both THSC20.HVTR04 and THSC20.HVTR26 mAb can bind to RBD in different stoichiometries both in the “up” and “down” RBD conformation on the spike **(Fig 6).** Next, we docked PDB 6VYB/6VXX into the density and labeled the N439, N440 and K444 residues as red spheres. For THSC20.HVTR04, these residues are directly in the epitope paratope interface, **S10 Fig**), explaining the reduction in neutralization when these residues are mutated. This observation corroborates with the X-ray structures as described above.

### THSC20.HVTR04 and THSC20.HVTR26 combination protects against Delta challenge in K18 hACE-2 mice

**Fig 7:**
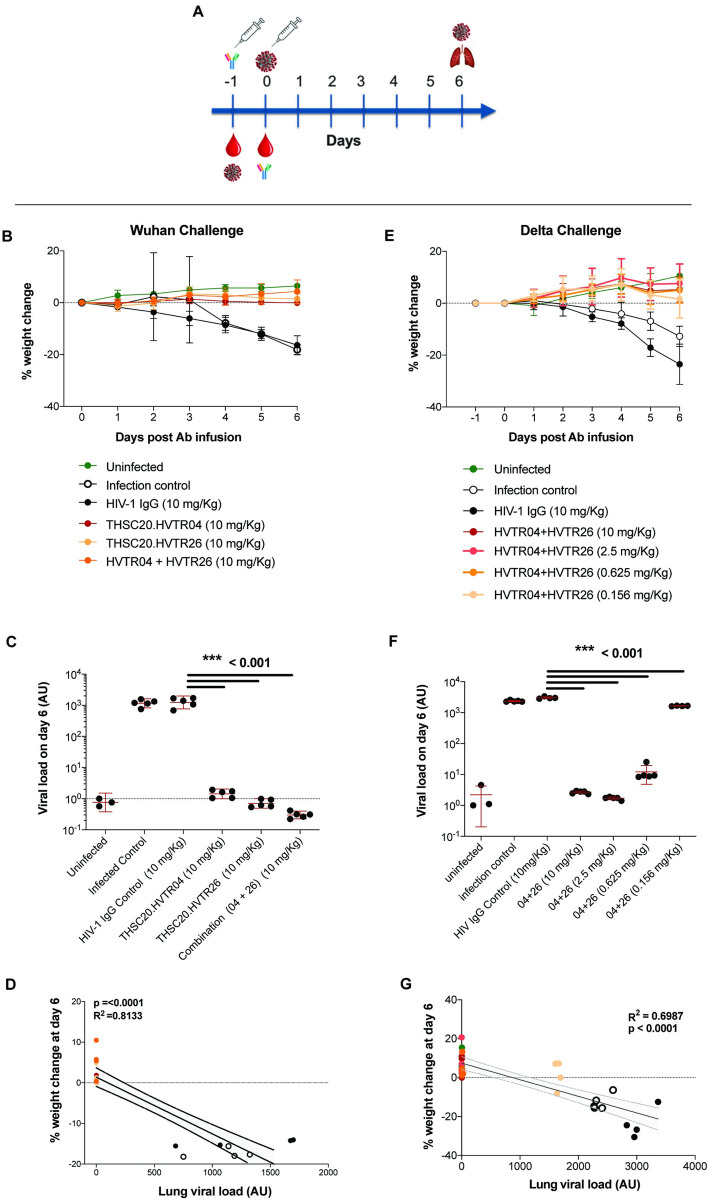
Neutralizing mAbs are able to protect against Wuhan and Delta variants in K18-hACE2 mice model. **A.** Experimental design of the efficacy assessment in K18-hACE2 transgenic mice model. K18-hACE2 mice were passively given intraperitoneal injection of the two individual mAbs or mixture of two mAbs 24 h prior to intranasal inoculation with 10^5^ PFU of SARS-CoV-2 Wuhan or Delta isolate. Each mouse was administered with single dose of 10 mg/kg body weight (equivalent to 200 ug /animal) for testing against Wuhan isolate and four different doses of 10 mg, 2.5 mg, 0.625 mg and 0.156 mg mAb per kg body weight (equivalent to 200μg, 50μg, 12.5μg and 3.125μg per animal respectively) for testing against Delta variant isolate. **B-D.** The prophylactic effect of THSC20.HVTR04 and THSC20.HVTR26 alone and in combination against Wuhan isolate on preventing body weight loss at concentration of 10 mg per kg body weight (**B**), lung viral load assessed at day 6 (**C**) and correlation of percent body weight change with lung viral load at day 6 (**D**). **E-G.** The prophylactic effect of THSC20.HVTR04 and THSC20.HVTR26 in combination against SARS-CoV-2 Delta variant on preventing body weight loss at four different concentrations as 10 mg, 2.5 mg, 0.625 mg and 0.156 mg mAb per kg body weight, day-wise and on day 6, respectively (**E**), Lung viral load assessed at day 6 (**F**), and correlation of percent body weight change with lung viral load at day 6 (**G**).

Based on their ability to neutralize SARS-CoV-2 variants with the highest potency, we selected THSC20.HVTR04 and THSC20.HVTR26 to assess protection efficacy against Wuhan and Delta variant challenge in a K18- human ACE2 (hACE2) transgenic mice model [36]. Moreover, both these mAbs were negative in an ELISA-based polyreactivity assay with solubilized CHO membrane proteins **(S11 Fig)**, further supporting their suitability for assessing their protective efficacy in the animal model. The experimental design of the efficacy assessment in K18-hACE2 transgenic mice model is shown in **Figs 7A and S12A**. We first assessed the protective efficacy of single doses of these two individual mAbs against SARS-CoV-2 (Wuhan isolate) infection in K18-hACE-2 transgenic mice **(Fig 7B–7D)**. A total of 28 mice were divided into six groups comprising five animals per group in the experimental arms and three animals in the Placebo group. Mice in groups 3, 4, 5 and 6 received intraperitoneal injection of 10 mg/kg of indicated antibodies (equivalent to 200 μg per animal) and were subsequently challenged intranasally with 10^5^ plaque forming units (PFUs) / mice of SARS-CoV-2 (Wuhan isolate) 24 hours after antibody administration. Prior to virus challenge, we obtained blood samples to determine serum IgG titers. The changes in body weight for all the experimental animals was measured daily until day 6 (end-point) **(Fig 7B)**. Additional clinical parameters were monitored to determine overall disease severity index at day 6–7 post challenge. All the mice were sacrificed on day 6 and lung tissues were harvested to measure lung virus load. As shown in **Fig 7B**, mice in the groups 2 (infection control) and 3 (non SARS-CoV-2 specific IgG control) exhibited significant weight loss of more than 10% on day 6 compared to those groups that received neutralizing antibodies. Moreover, mice that received neutralizing antibodies showed near undetectable viral RNA loads in their lung compared to the virus challenge group (group 2) the isotype control group (group 3) (P<0.001) **(Fig 7C)**. We observed a strong correlation (P<0.0001) between minimal weight loss and undetectable lung virus load in the mice that received neutralizing antibodies **(Fig 7D)**.

To further assess whether these two mAbs could also demonstrate protection against the more virulent Delta (B.1.617.2) variant, we repeated the same strategy as described above. For this, we arranged the mice into seven groups **(S12D Fig)** and titrated the combination of neutralizing antibodies from 10 mg/kg (equivalent to 200 μg per animal) to 0.156 mg/kg body weight (equivalent to 3.125μg per animal) through four-fold serial dilutions. The results are comparable to the Wuhan challenge experiment **(Figs 7E–7G and S8)**—all the mice in the infection control group (group 2) and the isotype control group (group 3) exhibited significant weight loss at day 6, whereas no significant weight loss was observed in animals that received the antibody cocktail including those receiving the lowest dose (0.156 mg/kg body weight; group 7) **(Fig 7E)**. In addition, as an indicator of clinical prognosis, we measured lung viral RNA at day 6 and found significant reduction in the lung viral load even at an antibody dose as low as 0.625mg/kg body weight (P<0.001) **(Fig 7F)**. Interestingly, we observed near 2-fold lower lung virus load in animals that received the lowest dose of the mAb cocktail (0.156 mg/kg body weight; group 7). As expected, a strong correlation between minimal or no loss in body weight and undetectable lung virus load in the mice that received different doses of mAb combination was observed **(Figs 7G and S12F)**. Taken together our study demonstrated significant protection by a combination of these two potently neutralizing mAbs at low dose against the highly virulent Delta variant in a transgenic hACE-2 mice model and was found to be comparable with few others tested against Delta variant [37,38].

## Discussion

Establishing antibody discovery capacity in low and middle-income countries (LMICs) is a key component of pandemic preparedness to enable prompt responses to emerging global health challenges. The likelihood of new virus variants emerging in LMIC is high as access to vaccines and treatment options are delayed or limited and there is an abundance of comorbidities that could accelerate the selection of new VOCs. Hence, enabling the discovery and development of monoclonal antibodies at the local level will provide new countermeasures that can be developed as treatment or prevention options that meet local needs while access to other interventions remain limited.

In our present study, we characterized mAbs that were isolated from an Indian convalescent donor in 2020 before the emergence of the deadly Delta variant. Among these mAbs, two (THSC20.HVTR04 and THSC20.HVTR26) showed neutralization of Alpha, Beta, Gamma and Delta VOCs and other VOIs including Kappa and Delta-Plus variants that were first detected in India. This pair of nAbs target non-competing epitopes on RBD and competed with RBD for binding to the ACE2 receptor on target cells. For THSC20.HVTR04, we identified residues N439, N440 and K444 within the RBM as critical for antibody neutralization by structural as well as experimental analyses. Of note, the ability of THSC20.HVTR04 to potently neutralize most VOCs could be explained by the fact that these mutants do not contain mutations at amino acid positions N439, N440 and K444 [39]. In contrast, the Omicron variant, which contains N440K and G446S mutations, is resistant to THSC20.HVTR04. Interestingly, two of the five newly isolated mAbs, THSC20.HVTR06 and THSC20.HVTR26 retained neutralization activity against Omicron variant albeit with reduced potencies. When compared with some of the existing mAbs developed for clinical use against SARS-CoV-2 by structural analysis, THSC20.HVTR04 resembles to some degree with Imdevimab and Bebtelovimab which were also shown to variably lose activity against Omicron [16]. However, THSC20.HVTR26 binds to SARS-CoV-2 RBD in a manner that makes it distinct to other clinically important mAbs. While a number of mAbs developed for clinical use completely lost activity against Omicron [16], two of the isolated mAbs reported in this study (THSC20.HVTR06 and THSC20.HVTR26) potently neutralized all the VOCs and VOIs examined and retained neutralization activity against the Omicron variant.

To evaluate *in vivo* activity, we assessed the prophylactic efficacy of our two best mAbs (THSC20.HVTR04 and THSC20.HVTR26) individually as well as in combination using an established model of SARS-CoV-2 infection in hACE2-expressing K18 transgenic mice [36]. Mice injected with THSC20-HVTR04 or THSC20-HVTR26 mAbs one day before Wuhan and Delta virus challenge were completely protected from weight loss and exhibited significant decrease in lung viral load compared to mice that were given HIV-1 mAb CAP256.VRC26.25 as an isotype control. Strikingly, efficacy assessment against Delta variant with four different doses of combination of two mAbs demonstrated complete protection of mice against Delta variant at dose of as low as 0.625 mg/kg body weight. These observations confirmed that the two best neutralizing mAbs assessed here conferred complete protection against SARS-CoV-2 Delta infection in this mice model at low antibody doses.

Our findings suggest that these two mAbs are valuable additions to the arsenal of existing potent mAbs against SARS-CoV-2 for development. The lessons learned from the unprecedented SARS-CoV-2 pandemic highlight the utility of timely response to such pandemics through discovery of effective mAbs against viral pathogens as one of the components of pandemic preparedness for combating coronaviruses and other deadly viruses. In the present scenario it is of paramount importance to promote and build capacity at LMICs to rapidly discover neutralizing mAbs against viral and other pathogens which will enable us to respond promptly to existing and future pandemics of coronaviruses or other highly transmissible viral pathogens.

## Methods

### Ethics statement

#### Ethics statement for use of human samples

The participants included in this study were members of DBT COVID-19 consortium cohort, organized by interdisciplinary research institutes and hospitals in the National Capital Region of India. It was coordinated by the Translational Health Science and Technology Institute. The main clinical sites were ESIC Medical College Hospital, Faridabad, and Loknayak Hospital, New Delhi. The study protocol was approved by the Institute Ethics Committees (IEC) of all participating institutions (IEC of ESIC Medical College Hospital, Faridabad, IEC of Loknayak Hospital, New Delhi and IEC of Translational Health Science & Technology Institute, Faridabad). Plasma and peripheral blood mononuclear cells (PBMCs) were prepared from blood samples obtained from eleven individuals between 6–8 weeks post recovery from SARS-CoV-2 infection who were infected in April 2020. The formal written consent was obtained from the participants.

#### Animals and ethics statement

Prior to the conduct of experiments to assess protective efficacy of the novel mAbs in small animals, approvals on the protocols involving dosing and animal challenge were obtained from the institutional animal ethics committee of the Translational Health Science & Technology Institute, Faridabad (approval # IAEC/THSTI/159), institutional biosafety committee of the Translational Health Science & Technology Institute, Faridabad (approval # 324/2021) and DBT Review Committee on Genetic Manipulation (RCGM; DBT RCGM approval #: IBKP UAC: TRARDSAB0214). 6–8 weeks old K18-hACE2 transgenic mice used to test the antibody efficacy were housed and maintained at the designated small animal facility (SAF) and subsequently transferred to the Animal biosafety level-3 (ABSL-3) institutional facility for infusion with mAbs and SARS-CoV2 challenge study. The animals were maintained under 12-hour light and dark cycle and fed with standard pellet diet and water *ad libitum*.

#### FACS sorting of antigen specific memory B cells

Antigen-specific single memory B cell sorting was performed in a FACS sorter (BD FACS Melody) essentially following the methods as described earlier [20]. Briefly, cryo-preserved PBMCs were first thawed at 37°C in a water bath and washed with an RPMI medium containing 10% fetal bovine sera (FBS) following incubation with fluorescently-labeled antibodies (BD Biosciences) against cell surface markers for CD3 (PE-Cy7); CD8 (PE-Cy7); CD14(PE-Cy7); CD16 (PE-Cy7); CD19 (BV421); CD20 (BV421); IgD (PerCP-Cy5.5); IgG (APC-H7) in addition to labelled RBD that was described elsewhere [20]as an antigen in FACS buffer containing PBS (pH7.4), 1% FBS, and 1.0 mM EDTA on ice. Live/Dead Fixable Aqua Blue Cell Stain (Thermo Fisher Inc.) was used to stain the cells for another 10 minutes on ice as per the manufacturer’s instructions. The avi-tagged SARS-CoV-2 RBD antigen was first labeled with biotin (Avidity, BirA500) was subsequently coupled to streptavidin-PE and streptavidin-APC (BD Biosciences) by incubating the mixture at 4°C for 1 hour at 4:1 molar ratio. The stained cells were subsequently washed with FACS buffer to remove unbound antibodies and probe and then filtered through 70-μm cell mesh (BD Biosciences) before processed in the FACS sorter. Single antigen RBD^+^CD3^-^CD8^-^CD14^-^CD16^-^CD19^+^CD20^+^IgD^-^IgG^+^ cells were sorted and collected into individual wells of a 96-well plate pre-filled with 20 ul of lysis buffer containing reverse transcriptase buffer (Thermo Fisher), IGEPAL (Sigma), DTT and RNAseOUT (Thermo Fisher). Plates containing sorted cells were sealed, snap-frozen on dry ice and stored at -80°C until used further.

#### Amplification and cloning of variable heavy and light IgG chains

cDNA Superscript III Reverse Transcription kit (Thermo Fisher) was used to prepare from sorted cells, cDNA master mix containing dNTPs, random hexamers, IgG gene-specific primers and RT enzyme was added to generate cDNA. Heavy and light-chain variable regions of IgG were amplified in independent nested PCR reactions using specific primers. First round PCR amplification was performed using HotStar Taq DNA Polymerases (Qiagen) and second round nested PCR was performed using Phusion HF DNA polymerase (Thermo Fisher Inc.). Specific restriction enzyme cutting sites (heavy chain, 5′-AgeI/3′-SalI; kappa chain, 5′-AgeI/3′-BsiWI; and lambda chain, 5′-AgeI/3′-XhoI) were introduced in the second round PCR primers in order to clone into the respective expression vectors. Amplified PCR products were verified on the agarose gel and wells with double positives (with amplification of both Heavy and Light chain variable regions from the same well) were identified and selected for subsequent cloning experiments. PCR products were digested with specific restriction enzymes, purified and cloned in-frame into expression vectors encoding the human IgG1, Ig kappa or Ig lambda constant domains using the Quick Ligase cloning system (New England BioLabs) according to the manufacturer instructions. Ligation reactions were transformed into NEB 5-alpha competent *E*. *coli* cells, plated on LB agar plates containing ampicillin and incubated overnight at 37oC in the incubator. Colonies with desired inserts were screened by colony PCR and used for preparation of plasmid DNA. Plasmid clones with correct insert were further confirmed by restriction digestion with respective (New England Biolabs, Inc.) restriction enzymes before being selected for the subsequent transfection experiment. Confirmed heavy and light chain plasmid DNA were co-transfected in 293T cells (ATCC) using Fugene transfection reagent (Promega) in 24 well plates for preparing antibody supernatant for initial screening for their expression and antigen specificity as detailed in the following section. Sanger sequencing were carried out to obtain the nucleotide and amino acid sequences of variable heavy and light IgG chains. Analysis of mAb sequences were carried out using the IMGT (www.imgt.org) V-quest webserver tool.

#### Capture ELISA for the detection of IgG expression

Maxisorp high protein binding 96 well ELISA plate (Nunc, Thermo Fisher Scientific.) was coated with 2μg/mL goat anti-human Fc antibody (Thermo Fisher Scientific) and incubated overnight at 4°C. Next day after washing, plates were blocked with 3% BSA in PBS (pH 7.4) for 1 hour at room temperature. After 3 times of washing with 1 X PBS containing 0.05% tween 20 (PBST), the cell supernatants which were harvested post transfection of antibody constructs in HEK 293T cells were added and incubated for 1 hour at room temperature. This was followed by addition of alkaline phosphatase-conjugated goat anti-human F(ab’)2 antibody (Thermo Fisher Scientific) Inc.) at 1:1000 dilution in 1% bovine serum albumin (BSA) incubated for an hour at room temperature. After the final wash, phosphatase substrate (Sigma-Aldrich Inc.) was added into the wells and absorption was measured at 405 nm on a 96 well microtiter plate reader.

#### Streptavidin ELISA for anti SARS- CoV-2 (RBD) antibody detection

2μg/mL of Streptavidin (G-Biosciences) was coated onto each wells of Nunc maxisorp high protein-binding 96 well ELISA plate (Thermo Fisher Scientific Inc.) and incubated overnight at 4°C. Next day after washing, plates were blocked with 3% BSA in PBS (pH 7.4) for 1 hour at room temperature. 2 μg/mL of Biotinylated—RBD protein was subsequently added and incubated the plate for 2 hours at room temperature. After washing the plates for three times with PBST, cell supernatants at various dilutions were added to the wells and the plate was further incubated for 1 hour at room temperature. Finally, HRP (horseradish peroxidase) conjugated anti-human IgG Fc secondary antibody was added at a dilution of 1:1000 containing 1% BSA and the plate was incubated for an hour at room temperature. After the final wash, TMB substrate (Thermo Fisher Scientific Inc.)) was added and subsequently 1N H_2_SO_4_ was added to stop the reaction. The absorption was measured at 450 nm.

#### Preparation and purification of IgG

The IgGs representing the mAbs were produced in either HEK 293T (ATCC) or Expi293 (Thermo Scientific) cells. Plasmid DNA expressing variable heavy and light IgG chains were transiently transfected into HEK293T or Expi293 cells using polyethylenimine (PEI). After 4–5 days of incubation, supernatants were harvested by centrifugation and filtered through a 0.2 μm membrane filter. Supernatants were then flowed slowly on to the Protein A (G Biosciences) beads in the column at 4°C in order to capture the secreted antibodies. Beads in the column were washed with five column volumes of 1X PBS at room temperature. Antibodies were eluted in two to three column volumes of 100 mM Glycine (pH 2.5) and immediately neutralized with 1M Tris-HCL (pH 8.0). Eluted antibodies were dialyzed using 10K MWCO SnakeSkin dialysis tubings (Thermo Fisher Scientific) against 1X PBS thrice and then concentrated in 30kDa NMWCO Amicon Ultra-15 Centrifugal Filter Units (Millipore). Antibody solutions were finally filtered through a 0.2 μm syringe filter (Thermo Fisher Scientific) before being used for further experiments. Concentration of IgG was measured by NanoDrop spectrophotometer and IgG heavy and light chain bands were visualized with 12% SDS PAGE analysis.

#### Quantitative RBD-ELISA

Anti-RBD IgG ELISA was performed essentially as described in Mehdi *et al*. [40]. For the screening of donors, plasma samples were three-fold diluted starting from 1:25 and were assessed for the presence of RBD binding IgG antibodies. To determine the mAb concentration in mice sera, a serial dilution of respective purified mAbs with known concentration was run as standard. mAb concentrations in mice sera were calculated for each sample dilution by interpolation of OD values from respective purified antibody dilutions using GraphPad Prism.

#### Microneutralization screening assay

Preliminary screening of heat-inactivated plasma samples obtained from convalescent donors for their neutralization potential were assessed as described in Malladi *et al*. [41] with slight modification. Briefly, plasma samples were serially two-fold diluted and mixed with 100 TCID_50_ of SARS-CoV-2 isolate. The virus-plasma mixture was transferred to Vero E6 monolayer seeded in 96 well plates in triplicate and incubated for 1 hour. The cell monolayer was subsequently washed with serum free media following which fresh complete medium was added. The plate was further incubated for 72 hours at 37°C in a humidified CO_2_ incubator. Absence of cytopathic effect (CPE) as an indicator of virus neutralization was assessed by observing the cells under a bright field microscope. The dilution at which no CPE was observed was considered as the neutralization titer.

#### Pseudovirus (PSV) neutralization assay

Pseudoviruses expressing complete SARS-CoV2 spike genes were prepared by transient transfection of HEK293T cells with three plasmids: SARS-CoV2 MLV-gag/pol and MLV-CMV-luciferase plasmids using Fugene 6 (Promega Inc.) as described earlier [20]. After 48-hour post transfection, cell supernatants containing pseudotyped viruses were harvested and frozen at -80°C until further use. Neutralization assay was carried out using HeLa-hACE2 cells for the infection of SARS-CoV-2 wild type and variant pseudoviruses. The purified IgGs were serially diluted and incubated with pseudoviruses in a humidified Incubator at 37^0^ C. After 1-hour incubation HeLa-hACE2 cells were added to the 96-well plates at 10,000 cells/well density. After 48 hours of incubation the luciferase activity was measured by adding Britelite substrate (Perkin Elmer Inc.) according to manufacturer’s instruction and RLU obtained using a luminometer (Victor X2, Perkin Elmer Inc).

#### Live virus focus-reduction neutralization test (FRNT)

The live virus neutralization assay was carried out following protocols as described by Bewley *et al*. [42]. Briefly, IgGs were serially diluted and incubated with indicated SARS-CoV-2 isolates. The virus-IgG mixtures were next added to Vero E6 cells for virus adsorption for one hour. The viral inoculum was removed, and cells were overlaid with carboxymethylcellulose and incubated for 24 hours. Cells were fixed and stained with anti-spike RBD antibody (Sino Biologicals) followed by HRP-conjugated anti-rabbit antibody (Invitrogen) and incubated with TrueBlue substrate (Sera Care). Finally, plates were washed with sterile MilliQ water, air-dried, and microplaques were quantified by AID iSPOT reader (AID GmbH, Strassberg, Germany). 50% neutralization values were calculated with four-parameter logistic regression using GraphPad Prism 7·0 software.

#### Cell surface spike binding assay

The binding of mAbs to the SARS-CoV-2 spikes expressed on the HEK 293T cell-surface was assessed as described previously with some modifications [20]. Briefly, HEK293T cells were transfected with the three plasmids used to generate SARS-CoV-2 pseudovirus (SARS-CoV-2 MLV-gag/pol, MLV-CMV-luciferase and SARS-CoV-2 spike plasmids). After incubation for 36–48 h at 37°C, cells were trypsinized and a single cell suspension was prepared which was distributed into 96-well U bottom plates. 3-fold serial dilutions of mAbs starting at 10 μg/ml and up to 0.041 μg/mL were prepared in 50 μl/well and added to the spike expressing as well as un-transfected 293T cells for 1 hour on ice. Cells were subsequently washed twice with FACS buffer (1x PBS, 2% FBS, 1 mM EDTA) and then stained with 50 μl/well of 1:200 dilution of R-Phycoerythrin AffiniPure F(ab’)₂ Fragment Goat Anti-Human IgG, F(ab’)₂ fragment specific antibody (Jackson ImmunoResearch Inc.) for 45 min. Cells were finally stained firstly with 1 LIVE/DEAD fixable aqua dead cell stain (ThermoFisher) in the same buffer for another 15 minutes and subsequently washed twice in plates with FACS buffer. The binding of mAbs to spikes expressing on cell surface was analyzed using flow cytometry (BD Canto Analyzer). Percent (%) PE-positive cells for antigen binding were calculated and the binding data were generated. CC12.1 (SARS-CoV-2 mAb), and CAP256.VRC26.25 antibody (HIV-1 bnAb) were used as positive and negative controls respectively for this experiment.

#### mAb-RBD competition assay

Inhibition of SARS-CoV-2 RBD binding by mAbs to the cell surface hACE2 was assessed by flow cytometry as described previously with some modifications [20]. Briefly, purified mAbs at 100 μg/mL and biotinylated SARS-CoV-2 RBD were mixed in 100 ul of DPBS in the molar ratio of 4:1 and incubated on ice for 1 hour. Parental HeLa and HeLa-ACE2 single cell suspension were prepared by washing cells once with DPBS and then detaching by incubation with DPBS supplemented with 5 mM EDTA. The detached HeLa and HeLa-ACE2 cell suspensions were again washed once and resuspended in FACS buffer (2% FBS and 1 mM EDTA in DPBS). 0.5 million Hela-ACE2 cells were added to the test mAb/RBD mixture and then incubated at 4°C for half an hour. 0.5 million HeLa and HeLa-ACE2 cells were incubated in separate wells with RBD alone without mAbs for use as background and positive control, respectively. After washing once with FACS buffer, HeLa and HeLa-ACE2 cells were resuspended in FACS buffer containing 1 μg/ml streptavidin-PE (BD Biosciences) and incubated for another half an hour. Cells were stained with 1:1000 final dilution of LIVE/DEAD fixable aqua dead cell stain (ThermoFisher) in the same buffer for another 15 minutes. HeLa and HeLa-ACE2 cells stained with SARS-CoV-2 RBD alone were used as background and positive control separately. The PE mean fluorescence intensity (MFI) was determined from the gate of singlet and live cells and the percentage of ACE2 binding inhibition was calculated by following formula.


$$100*(1-\frac{\mathrm{MFI}\;\mathrm{of}\;\mathrm{sample}-\mathrm{Average}\;\mathrm{MFI}\;\mathrm{of}\;\mathrm{background}}{\mathrm{Average}\;\mathrm{of}\;\mathrm{MFI}\;\mathrm{of}\;\mathrm{probe}\;\mathrm{alone}-\mathrm{Average}\;\mathrm{MFI}\;\mathrm{of}\;\mathrm{background}})$$

#### Biolayer interferometry for assessing RBD binding affinity

Streptavidin (SA) biosensors (Forte´ Bio) were used to assess the binding affinities of mAbs with SARS-CoV-2 RBD in PBST (PBS containing 0.02% Tween 20) at 30°C and 1,000 rpm. shaking on an Octet RED 98 instrument (Forte´ Bio Inc.). Sensors were first soaked in PBS for 15 minutes before being used to capture biotinylated SARS-CoV-2 RBD protein [20]. RBD was loaded to the biosensors up to a level of 1.0 nm. Biosensors were then immersed into PBS for 100 seconds and then immersed into wells containing specific concentrations of a mAb dissolved in PBST (PBS containing 0.02% Tween 20) for 500 seconds to measure association. A threefold dilution series with five different concentrations (33.3, 11.1, 3.7, 1.23, and 0.41 nM) was prepared for each mAb. Biosensors were next dipped into wells containing PBST for 500 seconds to measure dissociation. Data were reference- subtracted and aligned to each other using Octet Data Analysis software v10 (Forte´ Bio Inc.) based on a baseline measurement. Curve fitting was performed using a 1:1 binding model and data for all the five concentrations of mAbs. Kon, Koff and *K*D values were determined with a global fit.

#### Epitope binning

Epitope binning experiments were performed using Streptavidin (SA) biosensors (Forte´ Bio) and mAbs were binned into epitope specificities. All the incubation steps for binning experiments were performed in 1x PBS. 50–100 nM of biotinylated RBD protein antigens were loaded on streptavidin biosensors to achieve 0.9 to 1.3 nm of wavelength shift and then washed. Saturating concentration of mAbs (100μg/ml) was added for 10 min and competing mAbs at concentrations of 25 μg/ml were then added for 5 min in order to measure binding in the presence of saturating antibodies.

#### Site-directed mutagenesis

Point substitutions within RBD in SARS-CoV-2 spike gene were introduced by site-directed mutagenesis using the QuikChange II kit (Agilent Technologies Inc.) following the manufacturer’s protocol and by overlapping PCR strategy as described previously [43]. Successful incorporation of desired substitutions was confirmed by Sanger sequencing.

#### X-ray crystallography

THSC20.HVTR04 and THSC20.HVTR26 Fab domains were sub-cloned into an in-house cleavable Fab expression vector, expressed in Expi293 cells, and purified as above. Fab fragments were purified by gel filtration with equimolar amounts of SARS-CoV-2 RBD and concentrated to 10 mg/mL and 7 mg/mL respectively. THSC20.HVTR04 complexes were initially crystallized in 0.3 uL sitting drops with 50:50 mix of protein to crystallization reagent (1.5M ammonium sulfate, 12%(v/v) isopropanol, 0.1M imidazole/ hydrochloric acid pH 6.5) and optimized in 15 well hanging drop plates. Crystals were flash frozen with 10% glycerol or ethylene glycol as cryoprotectant. THSC20.HVTR26 complexes were similarly crystallized with 15% (w/v) PEG 20,000, 100 mM HEPES/ sodium hydroxide pH 7.0 as crystallization reagent. Data were collected at Diamond Light Source (UK) MX I04 using a wavelength of 0.9795 Å at ~100K and processed using their automated pipelines. The structure was solved in Phenix v1.19.2–4158, using search models from AlphaFold. Models were refined with hydrogens to minimize clashes in COOT v0.9.6 using 5–10% of the data as an R_free_ cross-validation test set. All structural images were generated with the PyMOL Molecular Graphics System v2.5.0 (Schrodinger LLC). These data have been deposited with PDB accession codes 7Z0X for THSC20.HVTR26-RBD complex and 7Z0Y for THSC20.HVTR04-RBD complex.

#### Negative stain EM analysis

A three-fold molar excess of Fab was incubated with SARS-2 CoV 6P Mut7 for 30 minutes at room temperature and deposited on a glow discharged carbon coated Cu grid. The complexes were stained with 2% uranyl formate (w/v) for 90 seconds. An FEI Tecnai Spirit at 120 keV paired with an FEI Eagle 4k x 4k CCD camera was used for data collection, and automated using the Leginon software [44]. Raw micrographs were stored in the Appion database [45]. Particles were picked with DogPicker [46] and data was processed in RELION 3.0 [47]. Figs were generated using UCSF Chimera [48].

#### Measuring antibody polyreactivity

The antibody polyreactivity was assessed as described earlier [20,49]. Solubilized CHO cell membrane protein (SMP) was coated onto 96-well half-area high-binding ELISA plates (Corning, 3690) at 5ug/mL in PBS overnight at 4°C. After washing, plates were blocked with PBS/3% BSA for 1 hour at room temperature (RT). Antibodies were diluted at 100ug/mL in 1% BSA with 5-fold serial dilution. Serially diluted samples were then added in plates and incubated for 1 hour at RT. After washing, alkaline phosphatase-conjugated goat anti-human IgG Fcy secondary antibody (Jackson ImmunoResearch, 109-055-008) was added in 1:1000 dilution and incubated for 1h at RT. After final wash, phosphatase substrate (Sigma-Aldrich, S0942-200TAB) was added into each well. The absorption was measured at 405 nm in a spectrophotometer. Den3 (Dengue-specific mAb) and Bococizumab (PCK9 antagonist) were included as benchmarking controls.

#### K18-hACE2 mice challenge

SARS-CoV-2 Wuhan (catalogue number: USA-WA1/2020) and B.1.617.2 delta (hCoV-19/USA/PHC658/2021 catalogue number NR-55611) were procured from BEI resources (https://www.beiresources.org/) and were expanded in Vero E6 cells to produce stocks required for the experiments. Mice randomly allotted to different groups (n = 5) *viz*, infection control and those received SARS-CoV-2 specific (THSC20.HVTR04 and THSC20.HVTR26) and non-specific (HIV CAP256.VRC26.25) monoclonal antibodies as IgG were housed in different cages. Antibody recipient groups were given intraperitoneal injection of IgG one day prior to challenge (day -1). Except for the unchallenged control group (n = 3), animals in all other groups were challenged with 10^5^ PFU of SARS-CoV2 (Wuhan and Delta isolates) intranasally on Day 0, administered through a catheter 25 μl/ nare under anesthesia by using ketamine (150mg/kg) and xylazine (10mg/kg) inside ABSL3 facility [36,50–52]. Unchallenged control group received mock PBS (pH 7.4) intranasally.

#### Gross clinical parameters of SARS-CoV-2 infection

All the infected animals were euthanized on day 6 days post infection at the ABSL3. Changes in body weight, activity of the animals were observed on each day post challenge. Post sacrifice, lungs of the animals were excised and imaged for gross morphological changes. Right lower lobe of the lung was fixed in 10% neutral formalin solution and used for histological analysis. Rest of the lung was homogenized in 2ml Trizol solution for viral load estimation. The tissue samples in trizol were stored immediately at -80°C till further use. Blood of the animals were drawn through retro-orbital vein on day -1 and 0 and through direct heart puncture at the end-point. Serum samples were stored at -80°C till further use.

#### Lung viral load quantification

Homogenized lung tissues were used for RNA isolation using Trizol-chloroform method as per the manufacturer’s protocol and quantitated in a Nanodrop. 1 μg of total RNA was then reverse-transcribed to cDNA using the iScript cDNA synthesis kit (Biorad; #1708891) (Roche). Diluted cDNAs (1:5) were used for qPCR by using KAPA SYBR FAST qPCR Master Mix (5X) Universal Kit (KK4600) on Fast 7500 Dx real-time PCR system (Applied Biosystems) and the results were analyzed with SDS2.1 software. The CDC-approved SARS-CoV-2 N gene primers: 5′-GACCCCAAAATCAGCGAAAT-3′ (Forward), 5′-TCTGGTTACTGCCAGTTGAATCTG-3′ (Reverse) were used for virus load estimation. The relative expression of each gene was expressed as fold change and was calculated by subtracting the cycling threshold (Ct) value of β-actin (endogenous control gene) from the Ct value of target gene (ΔCT). Fold change was then calculated according to the previously described formula POWER (2,-ΔCT) [53]. For absolute quantitation, known copy number of the virus RNA was used as a standard to generate the standard curve.

## Supporting information

S1 Table Convalescent donor history & background information. (DOCX)

S2 Table Heavy and light chain variable IgG sequence characteristics and germline usages. (DOCX)

S3 Table Neutralization potency of THSC20.HVTR04 and THSC20.HVTR26 and their combination against replication competent SARS-CoV-2 live VOCs. (DOCX)

S4 Table Neutralization breadth and potency of THSC20.HVTR04 and THSC20.HVTR26 and their combination against pseudoviruses expressing different SARS-CoV-2 VOC and VOI spike variants. (DOCX)

S5 Table Effect of key point mutations within RBM in SARS-CoV-2 spike on neutralization potency of THSC20.HVTR04 and THSC20.HVTR26 mAbs. (DOCX)

S6 Table X-ray crystallographic data collection and refinement statistics. (XLSX)

S1 Fig Representative Gating Strategy for the Antigen (RBD)-specific B cell sorting. **A.** Peripheral blood mononuclear cells (PBMCs) obtained from a convalescent donor C-03-0020 were stained with conjugated antibodies to cell surface markers, streptavidin labelled RBD probes and live dead stain and RBD-specific single B cells were sorted using a flow sorter. Singlet living CD19^+^C20^+^ IgG^+^ cells were gated and cells with positive SARS-CoV-2 RBD staining were selected for the single cell sorting into the 96 well plate prefilled with lysis buffer. **B.** There were 65.4% lymphocytes in total analyzed cells and among these 99.7% were single cells of which 98.8% (98.4% of total lymphocytes) were live cells. Of these 98.8% live cells, 6.01% (5.9% of total lymphocytes) were CD19+/CD20+ B cells; 6.25% of the CD19+/CD20+ B cells (0.37% of total lymphocytes) were IgG+ cells and 4.17% of the CD19+/CD20+/IgG+ cells (0.015% of total lymphocytes) were RBD+ CD19+/CD20+/IgG+ cells.
(PPTX)

S2 Fig Expression and antigenicity of the isolated mAbs. Supernatants harvested from HEK 293T cells co-transfected with IgG expression vectors were examined for expression by Fc-capture ELISA (black filled bar) and their ability to bind to SARS CoV2 receptor binding domain (RBD) used for B cell sorting by streptavidin ELISA (striped line bar). Non-specific IgG refers to an IgG that showed efficient expression but did not bind to RBD. Non-functional IgG refers to IgG sequence that neither expressed nor showed any RBD binding.
(PPTX)

S3 Fig Binding affinity and avidity of five monoclonal antibodies with SARS-CoV-2 spike RBD by BLI and ELISA. **A.** Binding affinities of THSC20.HVTR06, THSC20.HVTR39 and THSC20.HVTR88 to the SARS-CoV-2 (Wuhan) receptor binding domain (RBD) protein by BLI-Octet. Biotinylated wild type SARS-CoV-2 RBD antigen was immobilized on Streptavidin (SA) biosensors and binding affinity of monoclonal antibodies to RBD was tested using three-fold serial dilutions of mAbs starting with 33.3 nM and lowest 0.41 nM (five different concentrations were tested). Association and dissociation was assessed for 500 seconds each. Data shown is reference-subtracted and aligned using Octet Data Analysis software v11.1 (Forte Bio). Curve fitting was performed using a 1:1 binding model and K_on_, K_off_ and K_D_ values were determined with a global fit. **B.** Binding avidity of mAbs determined by RDB-ELISA. Four-fold serial dilutions of mAbs starting with 10ug/mL were tested for binding to RBD by ELISA. Data shown mean with SEM from two replicates from single experiment. EC50 values were obtained by curve fit method using GraphPad Prism.
(PPTX)

S4 Fig Comparison of neutralizing breadth and potency of all the mAbs isolated from the donor C-03-0020 in pseudovirus neutralization assay. Representative dose response curves from experiment with each concentration response tested in duplicate. THSC20HVTR04 and THSC20.HVTR26 mAbs were found to show maximum neutralization potency (lower panel, right) as determined by their IC50 values, obtained by non-linear regression four parameter curve fit method in GraphPad Prism. Shown values are mean with SEM.
(PPTX)

S5 Fig Live virus focus-reduction neutralization assay. The effect of combination of THSC20.HVTR04 and THSC20.HVTR26 was assessed by dose-dependent foci reduction neutralization (FRNT) live virus neutralization assay in Vero-E6 cells.
(PPTX)

S6 Fig Comparison of epitope specificities of the newly isolated mAbs by epitope binning. Biotinylated RBD was captured using streptavidin biosensor and indicated mAbs at a concentration of 100μg/ml first incubated for 10 min followed by incubation with 25μg/ml of competing antibodies for 5 min.
(PPTX)

S7 Fig Detailed analysis of structural insights of mAb bound to SARS-CoV-2 RBD. **A.** Schematic of THSC20.HVTR26 Fab (PDB 7Z0X, shown in dark and light green for heavy and light chains respectively) and THSC20.HVTR04 Fab (PBD 7Z0Y, shown in dark and light blue), bound to SARS-CoV-2 spike RBD (shown in purple). Human ACE2 bound to Omicron variant RBD (PDB 7WBP) is shown in yellow surface and cartoon view. Structures were aligned by RBD. **B.** Atomic details of the THSC20.HVTR26 interaction with RBD are shown, colored as in A, with sites mutated in the Omicron variant colored gold. The S477N_RBD_ mutation is not predicted to clash with THSC20.HVTR26 but may disrupt water-mediated bonds. The T478K_RBD_ mutation points directly into the Fab paratope, resulting in potential clashes (although this mutation does alone does not confer neutralization resistance as the Delta variant is still neutralized). **C.** Surface view of the THSC20.HVTR26 Fab paratope, colored as in A. SARS-CoV-2 spike residues 475–489 are shown in cartoon and stick view. The E484K mutation (Beta variant) is shown in cyan and retains contact surface. The E484A mutations (Omicron variant) is shown in pink and removes a third of the bound surface area at this position, contributing to neutralization resistance. **D.** The THSC20.HVTR26 interaction with RBD was modelled onto trimeric RBD-down spike protein. Fab bound protomer 1 was colored as in A, while adjacent protomers were colored white (RBD2) and yellow (RBD3). Mutations found in omicron are shown as pink spheres on adjacent protomer 2 (white) and may influence the conformation of the N343-linked glycan immediately adjacent to Fab1. **E.** Atomic details of the THSC20.HVTR04 interaction with RBD are shown, colored as in A, with sites mutated in the Omicron variant colored gold. The N440K_RBD_ substitution would disrupt hydrogen bonding with D49_LC_, and clash with CDR-L1, while G446S_RBD_ would clash with N56_LC_ and/or Y58_LC_ in CDR-L2. Q498R_RBD_ would also disrupt a water-mediated interaction with Y91_LC_ and G446_RBD_. Side chains were drawn in traditional ball-and-stick view, and key hydrogen bonds with N439_RBD_, N440_RBD_, and K444_RBD_ are indicated.
(TIF)

S8 Fig Surface representation of SARS-CoV-2 RBD (purple) bound to THSC20.HVTR04 and THSC20.HVTR26 in comparison to current clinically important mAbs. **A.** The epitopes for neutralizing antibodies THSC20.HVTR04 (this manuscript, PDB 7Z0Y, blue), Imdevimab (REGEN-COV / Ronapreve, PBD 6XDG, light green), Bebtelovimab (LY-CoV1404, PDB 7MMO, brown), Cilgavimab (Evusheld, PDB 7L7E, white), and Sotrovimab (S309, PDB 7TN0, red) were calculated as contact sites within 5 Å. **B.** The epitopes for neutralizing antibodies THSC20.HVTR26 (this manuscript, PDB 7Z0X, green), Tixagevimab (Evusheld, PDB 7L7D, black), Bamlanivimab (LY-CoV555, PDB 7KMG, beige), Regdanvimab (CT-P59, PDB 7CM4, yellow), Casirivimab (REGEN-COV / Ronapreve, PBD 6XDG,pink), Amubarvimab (P2C-1F11 / BRII-196, PDB 7CDI, teal green), and Etesevimab (LY-CoV016, PDB 7C01, cyan) are shown, as in A. **C.** Primary sequence of SARS-CoV-2 RBD positions 410 to 510 (purple), and mutations associated with the Beta (cyan), Delta+ (lime), or Omicron (salmon) variants. Contact positions for several clinically relevant neutralizing antibodies are shown with dots, calculated and colored as in A or B.
(TIF)

S9 Fig 2D class averages of NS-EM of mAb-spike complexes. Low resolution images of the SEC purified complex of THSC20.HVTR04 and THSC20.HVTR26 Fabs with SARS-CoV-2 spike protein which were subsequently used for further refinement to generate 3D reconstruction shown in Fig 6.
(PPTX)

S10 Fig Close-up view of the epitope-paratope interface of SARS-2 CoV 6P Mut7 in complex with Fabs THSC20.HVTR04 and THSC20.HVTR26. PDB 6VYB and a polyalanine Fab model fit into the spike and fab nsEM densities, respectively.
(PDF)

S11 Fig Poly-reactivity assessment of isolated mAbs. The polyreactivity of THSC20.HVTR04 and THSC20.HVTR26 mAbs using CHO soluble membrane protein (SMP) by ELISA. Three-fold serial dilutions of mAbs starting with 100ug/mL were tested.
(PPTX)

S12 Fig Dose-response effect of mAbs given singly and in combination on protection of mice against Wuhan and Delta infections. A. Animal grouping and antibody doses given (Wuhan challenge). B. Comparison of body weights between animals that received antibodies and those who did not prior to challenge with Wuhan isolate. C. Quantification of circulating serum IgG in mice at day 0 one day after infusion of mAbs at indicated doses and before virus (Wuhan) challenge. Values represent mean with SEM. D. Animal grouping and different antibody combination doses given (Delta challenge). E. Percent change in body weight of animals that received different doses of mAb combinations. Values represent mean with SEM. F. Quantification of circulating serum IgG concentration in mice at day 0 one day after infusion of mAbs at indicated doses and before virus (Delta) challenge. Values represent mean with SEM. G. Correlation between percent body weight change on day 6, circulating serum IgG concentration on day 0 and lung viral load on day 6 in mice those received different concentrations of mAb combinations.
(PPTX)
