# Supplementary material for: A combination of potently neutralizing monoclonal antibodies isolated from an Indian convalescent donor protects against the SARS-CoV-2 Delta variant
Source: PLoS Pathog. 2022 Apr 28;18(4):e1010465. doi: 10.1371/journal.ppat.1010465 (PMC9089897; doi:10.1371/journal.ppat.1010465)
Supplement: S1 Table — (DOCX) [file ppat.1010465.s001.docx]

**Table S1**. Background information of convalescent donors.

| **PID** | **Age** | **Gender** | **Month & Year of infection** | **Clinical status at the time of blood draw** | **Blood drawn (Days post onset of detection)** | **Disease severity** |  |
| --- | --- | --- | --- | --- | --- | --- | --- |
| C-03-0008 | 56 | Female | April, 2020 | Symptomatic | 55 | Mild/Moderate |  |
| C-03-0015 | 46 | Male | April, 2020 | Asymptomatic | 47 | None |  |
| C-03-0020 | 33 | Male | April, 2020 | Symptomatic | 57 | Mild/Moderate |  |
| C-09-0001 | 30 | Male | April, 2020 | Asymptomatic | 42 | None |  |
| C-09-0002 | 44 | Male | April, 2020 | Symptomatic | 50 | Mild/Moderate |  |
| C-09-0003 | 51 | Male | April, 2020 | Symptomatic | 56 | Mild/Moderate |  |
| C-09-0004 | 50 | Female | April, 2020 | Symptomatic | 53 | Mild/Moderate |  |
| C-10-0006 | 24 | Male | April, 2020 | Asymptomatic | 40 | None |  |
| C-10-0009 | 42 | Male | April, 2020 | Asymptomatic | 39 | None |  |
| C-13-0009 | 67 | Male | April, 2020 | Symptomatic | 50 | Mild/Moderate |  |
| C-13-0022 | 45 | Male | April, 2020 | Symptomatic | 47 | Mild/Moderate |  |
