# Supplementary material for: A combination of potently neutralizing monoclonal antibodies isolated from an Indian convalescent donor protects against the SARS-CoV-2 Delta variant
Source: PLoS Pathog. 2022 Apr 28;18(4):e1010465. doi: 10.1371/journal.ppat.1010465 (PMC9089897; doi:10.1371/journal.ppat.1010465)
Supplement: S2 Table — (DOCX) [file ppat.1010465.s002.docx]

**Table 2.** Heavy and light chain variable IgG sequence characteristics and germline usages**.**

Heavy chain IgG


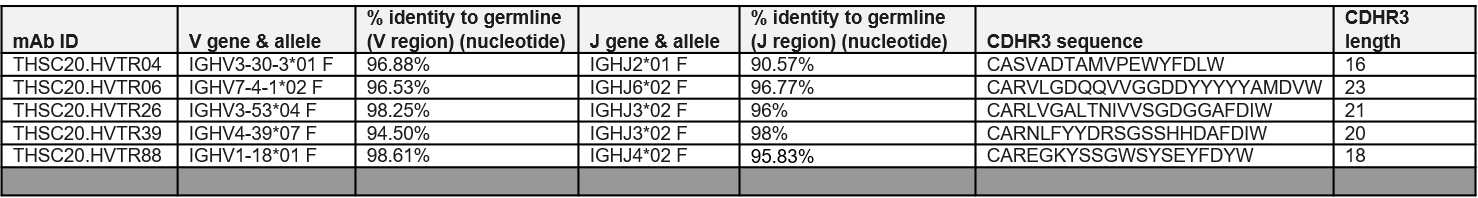


Light chain IgG


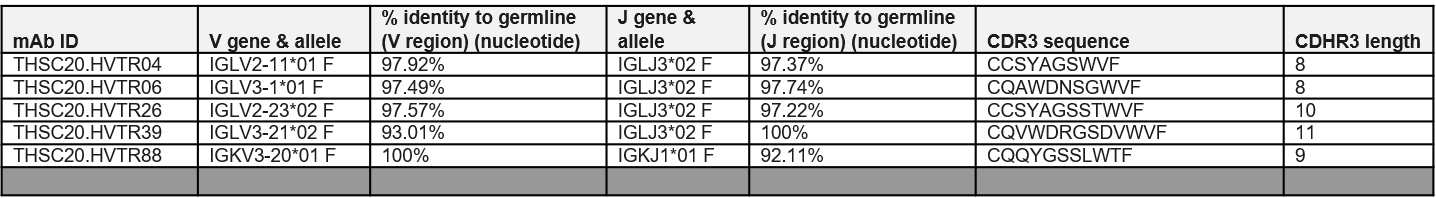


*The analysis of variable heavy (HC) and light (LC) chains IgG nucleotide sequences of the mAbs were done using by IMGT/V-QUEST tool (www.imgt.org).*
