# Supplementary material for: A combination of potently neutralizing monoclonal antibodies isolated from an Indian convalescent donor protects against the SARS-CoV-2 Delta variant
Source: PLoS Pathog. 2022 Apr 28;18(4):e1010465. doi: 10.1371/journal.ppat.1010465 (PMC9089897; doi:10.1371/journal.ppat.1010465)
Supplement: S3 Table — (DOCX) [file ppat.1010465.s003.docx]

**Table S3.** Neutralization potency of THSC20.HVTR04 and THSC20.HVTR26 and their combination against replication competent SARS-CoV-2 live VOCs.

|  | **IC50 (µg/mL)** | | | |
| --- | --- | --- | --- | --- |
|  | THSC20.HVTR04 | THSC20.HVTR26 | THSC20.HVTR 04+ 26 | CC12.3 |
| SARS-CoV-2 | 0.01 | 0.009 | 0.011 | 0.034 |
| B.1.1.7 (alpha) | 0.005 | 0.006 | 0.007 | 0.037 |
| B.1.351 (beta) | 0.006 | 0.014 | 0.007 | >20 |
| B.1.617.1 (kappa) | 0.008 | 0.011 | 0.011 | 0.044 |
| B.1.617.2 (delta) | 0.003 | 0.007 | 0.006 | 0.013 |

*Live virus focus reduction neutralization assay was carried out in Vero-E6 cells. Dose-dependent neutralization of SARS-CoV-2 variants by mAbs and their combination was measured to obtain mAb potencies expressed as µg/mL).*
