# Supplementary material for: A combination of potently neutralizing monoclonal antibodies isolated from an Indian convalescent donor protects against the SARS-CoV-2 Delta variant
Source: PLoS Pathog. 2022 Apr 28;18(4):e1010465. doi: 10.1371/journal.ppat.1010465 (PMC9089897; doi:10.1371/journal.ppat.1010465)
Supplement: S4 Table — (DOCX) [file ppat.1010465.s004.docx]

**Table S4.** Neutralization breadth and potency of THSC20.HVTR04 and THSC20.HVTR26 and their combination against pseudoviruses expressing different SARS-CoV-2 VOC and VOI spike variants.

|  | **IC50 (µg/mL)** | | | | | | |
| --- | --- | --- | --- | --- | --- | --- | --- |
|  | THSC20.HVTR04 | THSC20.HVTR26 | THSC20.HVTR 04+ 26 | REGN10933 | REGN10987 | CC6.30 | CC12.1 |
| SARS-CoV-1 | >20 | >20 | >20 | >20 | >20 | >20 | >20 |
| SARS-CoV-2 | 0.002 | 0.035 | 0.084 | 0.027 | 0.067 | 0.064 | 0.036 |
| B.1.1.7 (alpha) | 0.002 | 0.009 | 0.017 | 0.004 | 0.003 | 0.026 | 0.140 |
| B.1.351 (beta) | 0.001 | 0.004 | 0.041 | 2.357 | 0.009 | >20 | >20 |
| P.1 (gamma) | 0.002 | 0.001 | 0.001 | >20 | 0.007 | >20 | >20 |
| B.1.617.1 (kappa) | 0.003 | 0.009 | 0.049 | 0.061 | 0.071 | >20 | 0.025 |
| B.1.617.2 (delta) | 0.002 | 0.018 | 0.081 | 0.003 | 0.079 | 0.054 | 0.034 |
| Delta plus | 0.008 | 0.011 | 0.078 | 0.014 | 0.270 | >20 | >20 |

*Pseudovirus neutralization assay was carried out in HeLa-ACE2 cell line and neutralization was determined by measuring reduction of luciferase activity. Dose-dependent neutralization of SARS-CoV-2 variants by mAbs and their combination was measured to obtain mAb potencies expressed as µg/mL).*
