# Supplementary material for: A combination of potently neutralizing monoclonal antibodies isolated from an Indian convalescent donor protects against the SARS-CoV-2 Delta variant
Source: PLoS Pathog. 2022 Apr 28;18(4):e1010465. doi: 10.1371/journal.ppat.1010465 (PMC9089897; doi:10.1371/journal.ppat.1010465)
Supplement: S5 Table — (DOCX) [file ppat.1010465.s005.docx]

**Table S5.** Effect of key point mutations within RBM in SARS-CoV-2 spike on neutralization potency of THSC20.HVTR04 and THSC20.HVTR26 mAbs.

|  |  | **THSC20.HVTR04** | | **THSC20.HVTR26** | | |
| --- | --- | --- | --- | --- | --- | --- |
| ***Mutations*** | ***Region*** | ***IC_50_*** | ***Fold reduction****^§^* | ***IC_50_*** | ***Fold reduction****^§^* |  |
| WT (SARS-CoV-2) | - | 0.002 | - | 0.03 | - |  |
| R346K | RBD | 0.005 | 2.5 | 0.06 | 2 |  |
| K417N | RBD | 0.0015 | 0.75 | 0.05 | 1.66 |  |
| N439K | RBM | 0.3244 | 162.2 | 0.02 | 0.66 |  |
| N440K | RBM | 0.05 | 25 | 0.03 | 1 |  |
| K444N | RBM | 0.022 | 11 | 0.04 | 1.33 |  |
| F486L | RBM | 0.003 | 1.5 | 0.019 | 0.63 |  |
| F490S | RBM | 0.004 | 2 | 0.01 | 0.33 |  |
| N440K+D614G (B.1.36) | RBM/RBD | 0.182 | 91 | 0.02 | 0.66 |  |

*IC_50_ values are given as µg/mL and were assessed in pseudovirus neutralization assay.*

*^§^ Values represent fold reduction in IC50 values when compared to the wild type (WT) SARS-CoV-2 spike in pseudovirus neutralization assay.*
