# Supplementary figures and images for: A combination of potently neutralizing monoclonal antibodies isolated from an Indian convalescent donor protects against the SARS-CoV-2 Delta variant
Source: PLoS Pathog. 2022 Apr 28;18(4):e1010465. doi: 10.1371/journal.ppat.1010465 (PMC9089897; doi:10.1371/journal.ppat.1010465)

## Slide 1
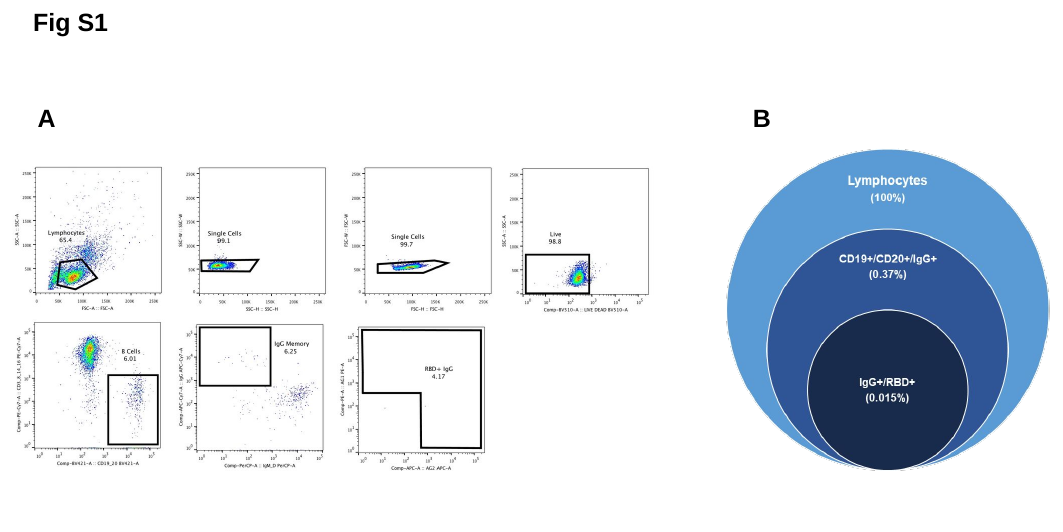

Fig S1
A
B

Supplement: S1 Fig — A. Peripheral blood mononuclear cells (PBMCs) obtained from a convalescent donor C-03-0020 were stained with conjugated antibodies to cell surface markers, streptavidin labelled RBD probes and live dead stain and RBD-specific single B cells were sorted using a flow sorter. Singlet living CD19+C20+ IgG+ cells were gated and cells with positive SARS-CoV-2 RBD staining were selected for the single cell sorting into the 96 well plate prefilled with lysis buffer. B. There were 65.4% lymphocytes in total analyzed cells and among these 99.7% were single cells of which 98.8% (98.4% of total lymphocytes) were live cells. Of these 98.8% live cells, 6.01% (5.9% of total lymphocytes) were CD19+/CD20+ B cells; 6.25% of the CD19+/CD20+ B cells (0.37% of total lymphocytes) were IgG+ cells and 4.17% of the CD19+/CD20+/IgG+ cells (0.015% of total lymphocytes) were RBD+ CD19+/CD20+/IgG+ cells. (PPTX) [file ppat.1010465.s007.pptx]

## Slide 1
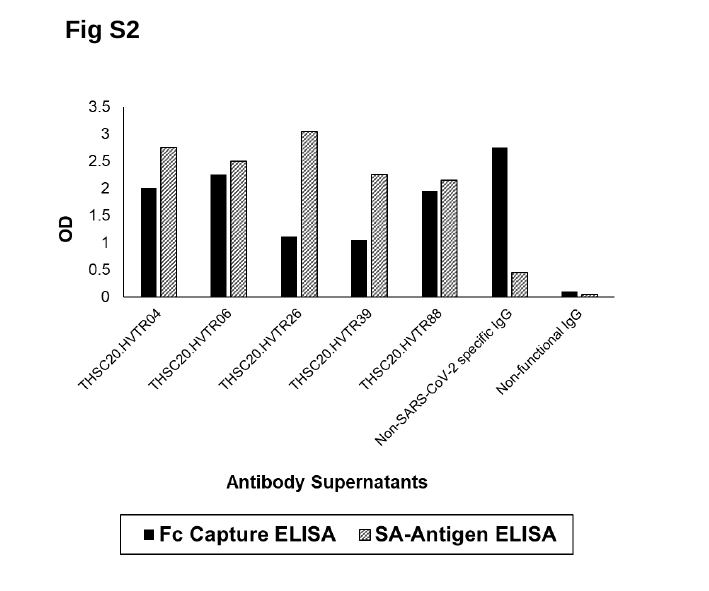

Fig S2

Supplement: S2 Fig — Supernatants harvested from HEK 293T cells co-transfected with IgG expression vectors were examined for expression by Fc-capture ELISA (black filled bar) and their ability to bind to SARS CoV2 receptor binding domain (RBD) used for B cell sorting by streptavidin ELISA (striped line bar). Non-specific IgG refers to an IgG that showed efficient expression but did not bind to RBD. Non-functional IgG refers to IgG sequence that neither expressed nor showed any RBD binding. (PPTX) [file ppat.1010465.s008.pptx]

## Slide 1
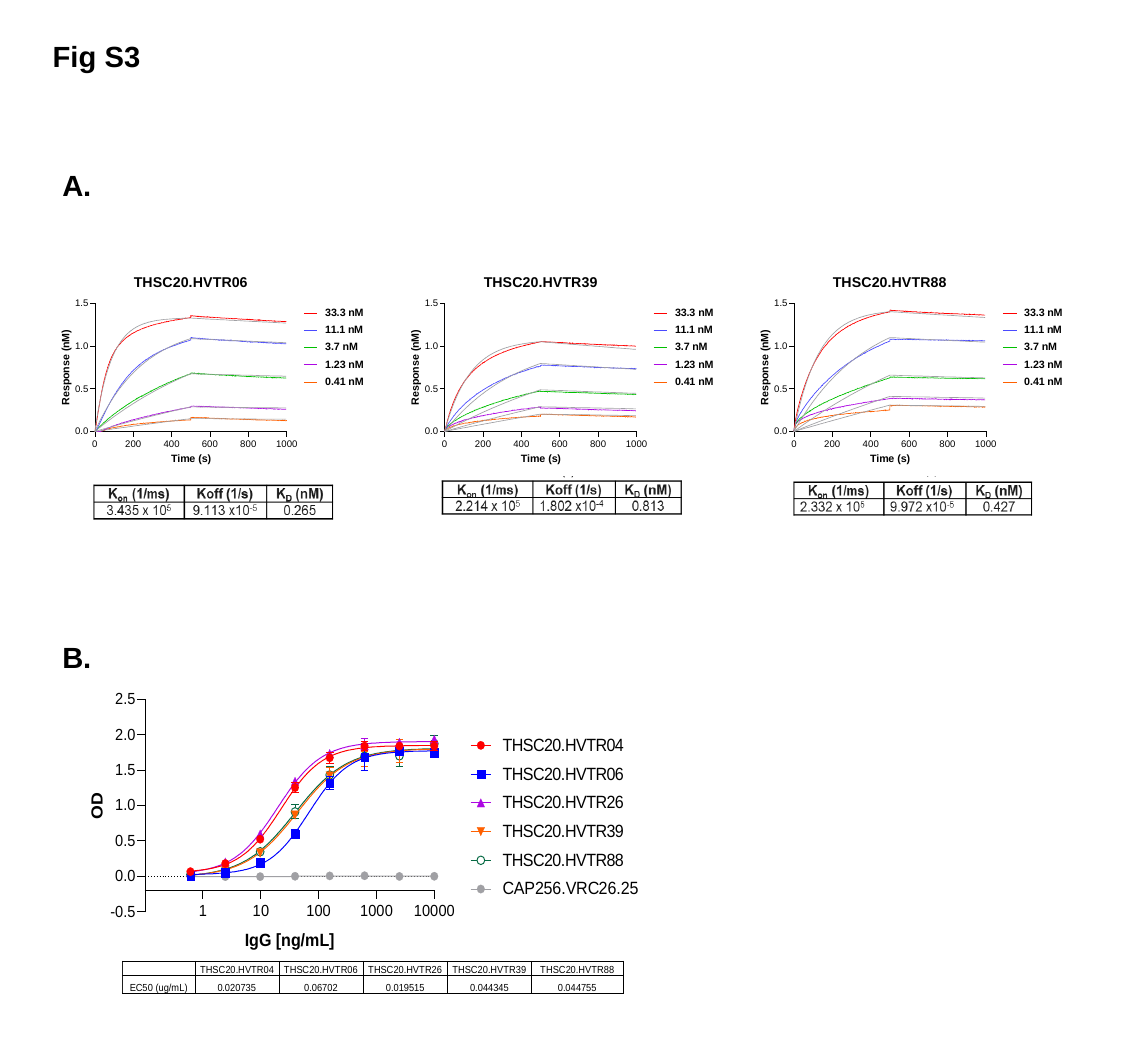

Fig S3
A.
B.

Supplement: S3 Fig — A. Binding affinities of THSC20.HVTR06, THSC20.HVTR39 and THSC20.HVTR88 to the SARS-CoV-2 (Wuhan) receptor binding domain (RBD) protein by BLI-Octet. Biotinylated wild type SARS-CoV-2 RBD antigen was immobilized on Streptavidin (SA) biosensors and binding affinity of monoclonal antibodies to RBD was tested using three-fold serial dilutions of mAbs starting with 33.3 nM and lowest 0.41 nM (five different concentrations were tested). Association and dissociation was assessed for 500 seconds each. Data shown is reference-subtracted and aligned using Octet Data Analysis software v11.1 (Forte Bio). Curve fitting was performed using a 1:1 binding model and Kon, Koff and KD values were determined with a global fit. B. Binding avidity of mAbs determined by RDB-ELISA. Four-fold serial dilutions of mAbs starting with 10ug/mL were tested for binding to RBD by ELISA. Data shown mean with SEM from two replicates from single experiment. EC50 values were obtained by curve fit method using GraphPad Prism. (PPTX) [file ppat.1010465.s009.pptx]

## Slide 1
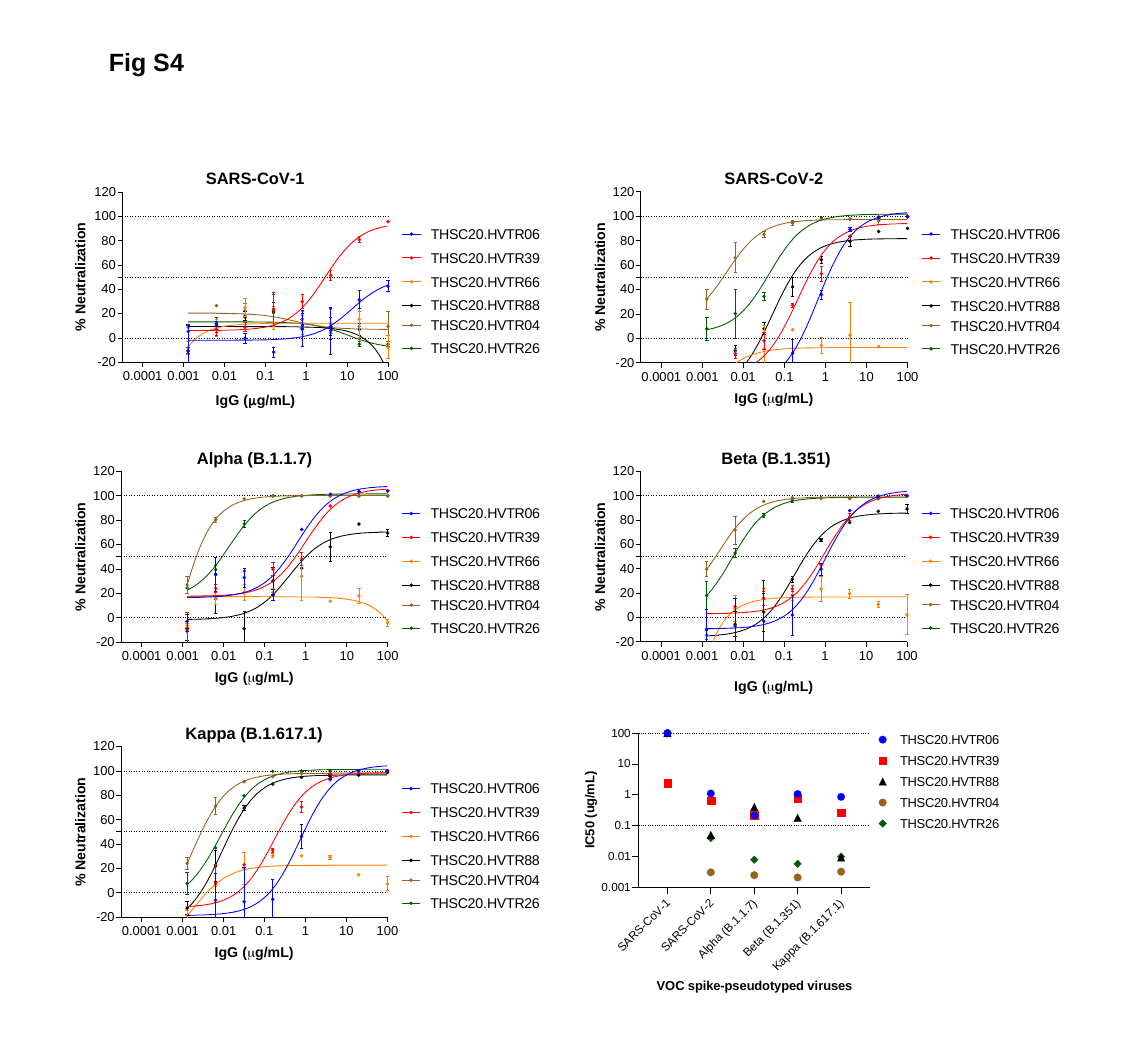

Fig S4

Supplement: S4 Fig — Representative dose response curves from experiment with each concentration response tested in duplicate. THSC20HVTR04 and THSC20.HVTR26 mAbs were found to show maximum neutralization potency (lower panel, right) as determined by their IC50 values, obtained by non-linear regression four parameter curve fit method in GraphPad Prism. Shown values are mean with SEM. (PPTX) [file ppat.1010465.s010.pptx]

## Slide 1
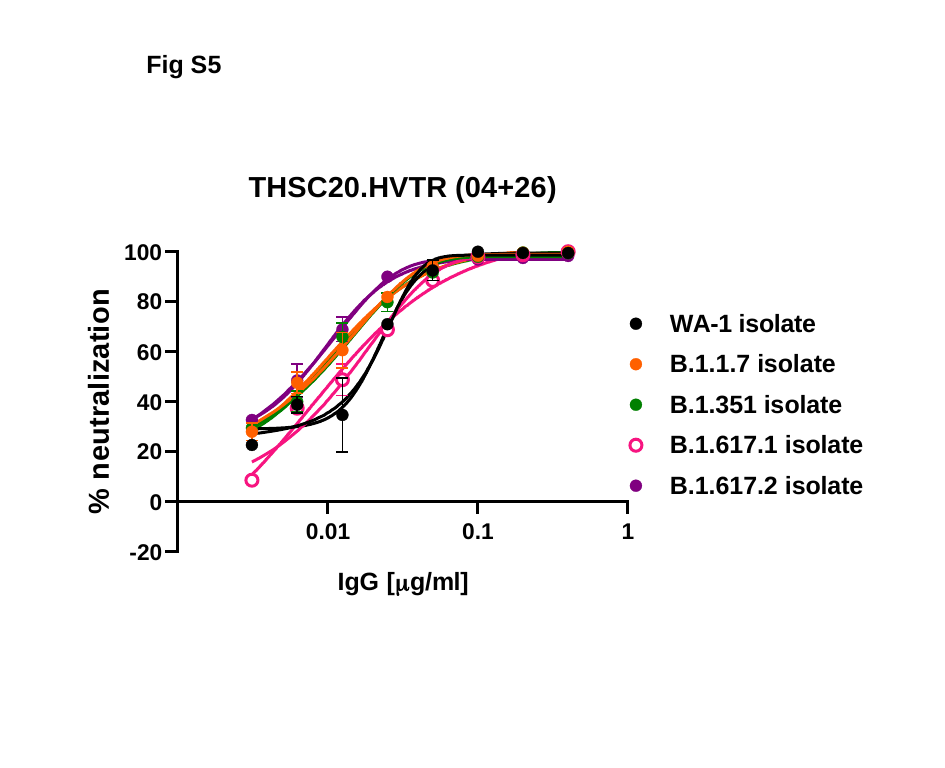

Fig S5

Supplement: S5 Fig — The effect of combination of THSC20.HVTR04 and THSC20.HVTR26 was assessed by dose-dependent foci reduction neutralization (FRNT) live virus neutralization assay in Vero-E6 cells. (PPTX) [file ppat.1010465.s011.pptx]

## Slide 1
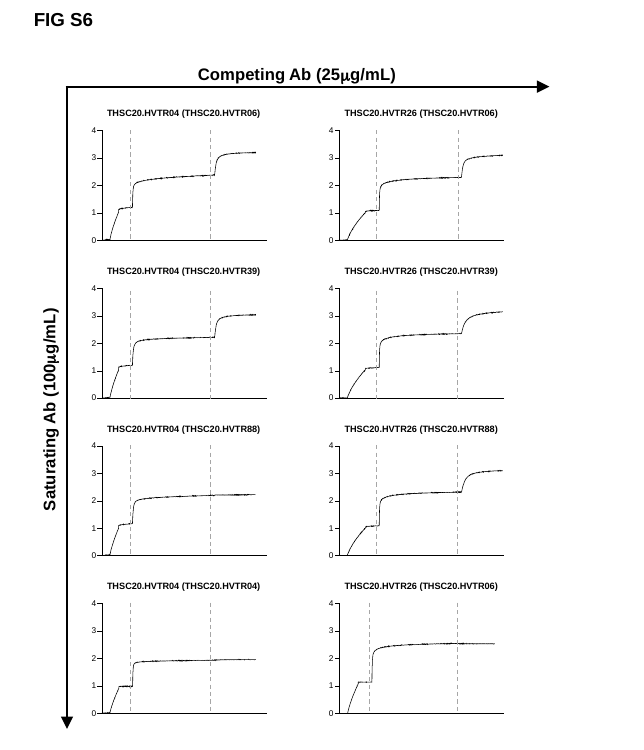

FIG S6
Competing Ab (25mg/mL)
Saturating Ab (100mg/mL)

Supplement: S6 Fig — Biotinylated RBD was captured using streptavidin biosensor and indicated mAbs at a concentration of 100μg/ml first incubated for 10 min followed by incubation with 25μg/ml of competing antibodies for 5 min. (PPTX) [file ppat.1010465.s012.pptx]

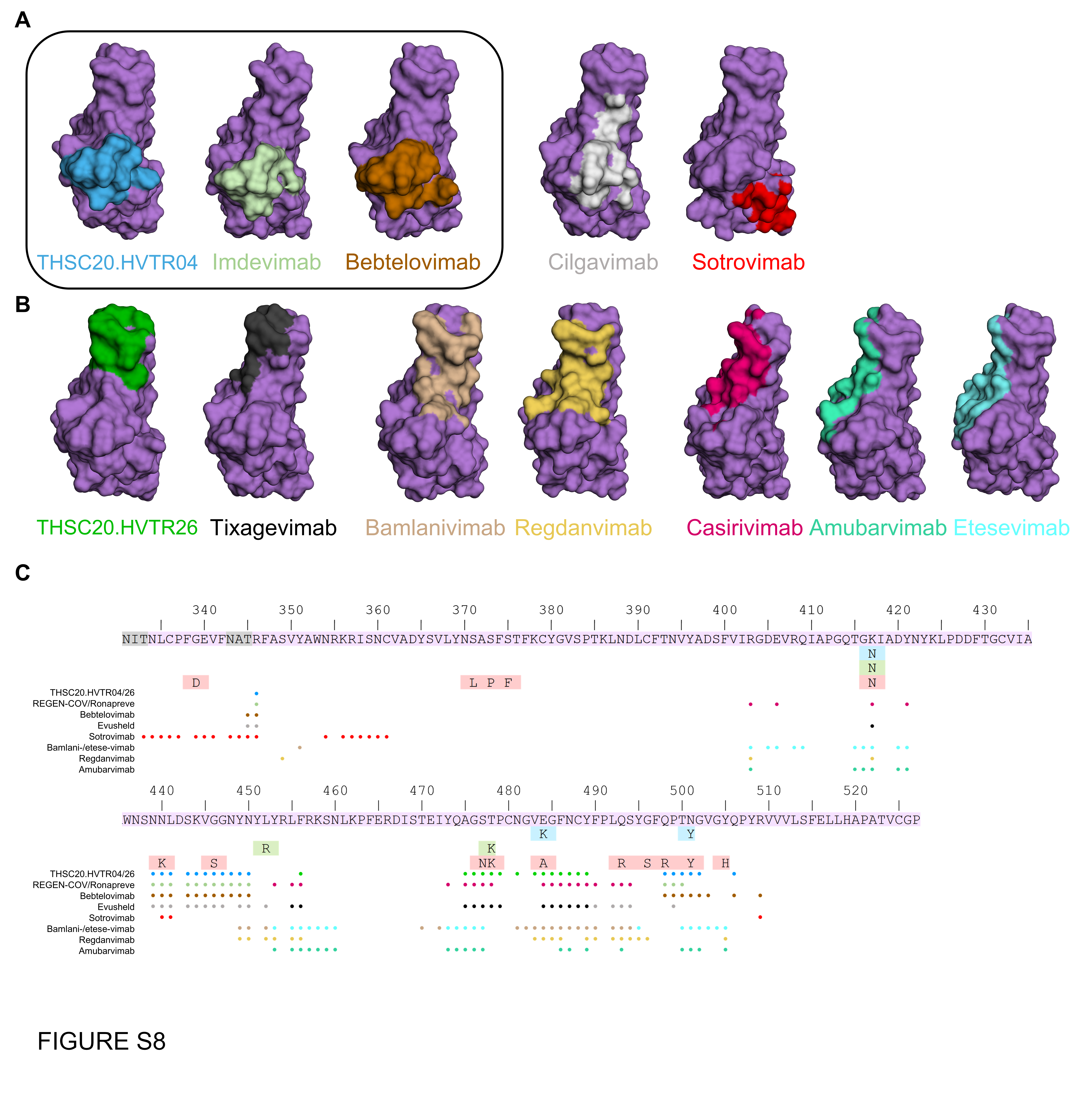

Supplement: S8 Fig — A. The epitopes for neutralizing antibodies THSC20.HVTR04 (this manuscript, PDB 7Z0Y, blue), Imdevimab (REGEN-COV / Ronapreve, PBD 6XDG, light green), Bebtelovimab (LY-CoV1404, PDB 7MMO, brown), Cilgavimab (Evusheld, PDB 7L7E, white), and Sotrovimab (S309, PDB 7TN0, red) were calculated as contact sites within 5 Å. B. The epitopes for neutralizing antibodies THSC20.HVTR26 (this manuscript, PDB 7Z0X, green), Tixagevimab (Evusheld, PDB 7L7D, black), Bamlanivimab (LY-CoV555, PDB 7KMG, beige), Regdanvimab (CT-P59, PDB 7CM4, yellow), Casirivimab (REGEN-COV / Ronapreve, PBD 6XDG,pink), Amubarvimab (P2C-1F11 / BRII-196, PDB 7CDI, teal green), and Etesevimab (LY-CoV016, PDB 7C01, cyan) are shown, as in A. C. Primary sequence of SARS-CoV-2 RBD positions 410 to 510 (purple), and mutations associated with the Beta (cyan), Delta+ (lime), or Omicron (salmon) variants. Contact positions for several clinically relevant neutralizing antibodies are shown with dots, calculated and colored as in A or B. (TIF) [file ppat.1010465.s014.tif]

## Slide 1
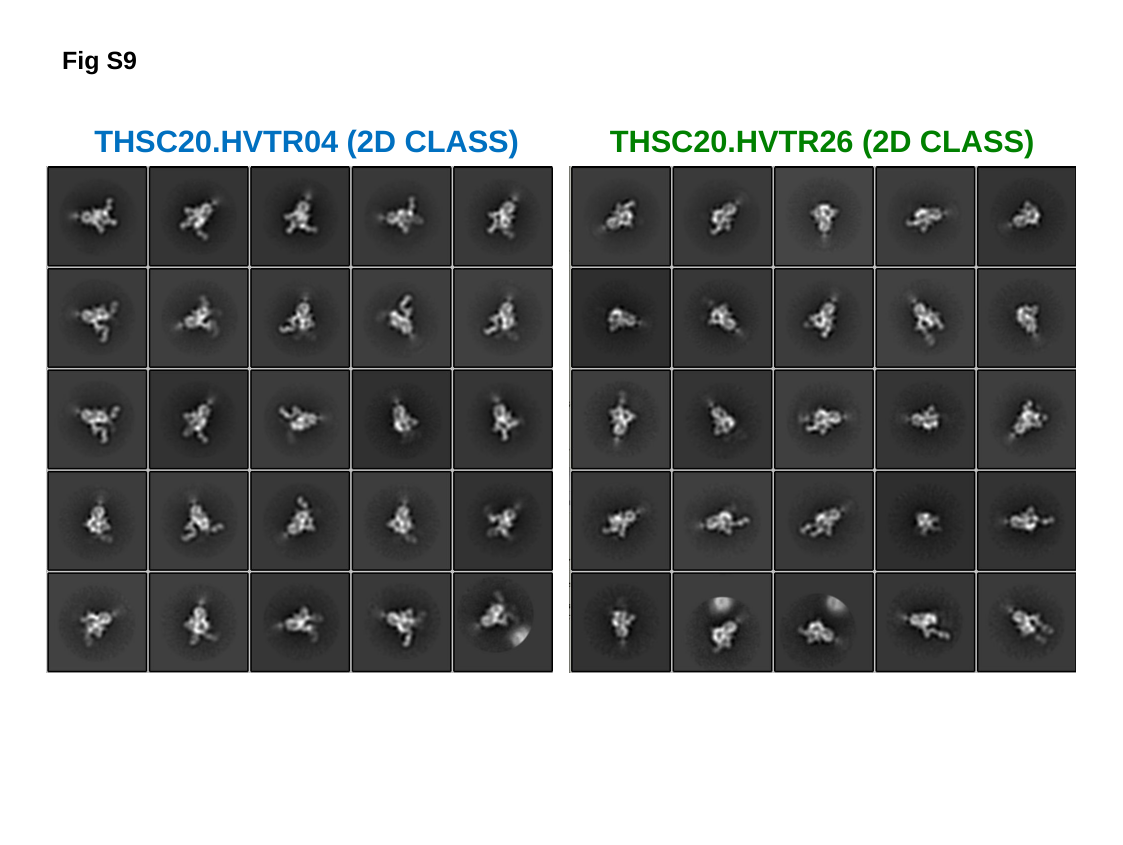

Fig S9
THSC20.HVTR04 (2D CLASS)
THSC20.HVTR26 (2D CLASS)

Supplement: S9 Fig — Low resolution images of the SEC purified complex of THSC20.HVTR04 and THSC20.HVTR26 Fabs with SARS-CoV-2 spike protein which were subsequently used for further refinement to generate 3D reconstruction shown in Fig 6. (PPTX) [file ppat.1010465.s015.pptx]

**Figure S10**

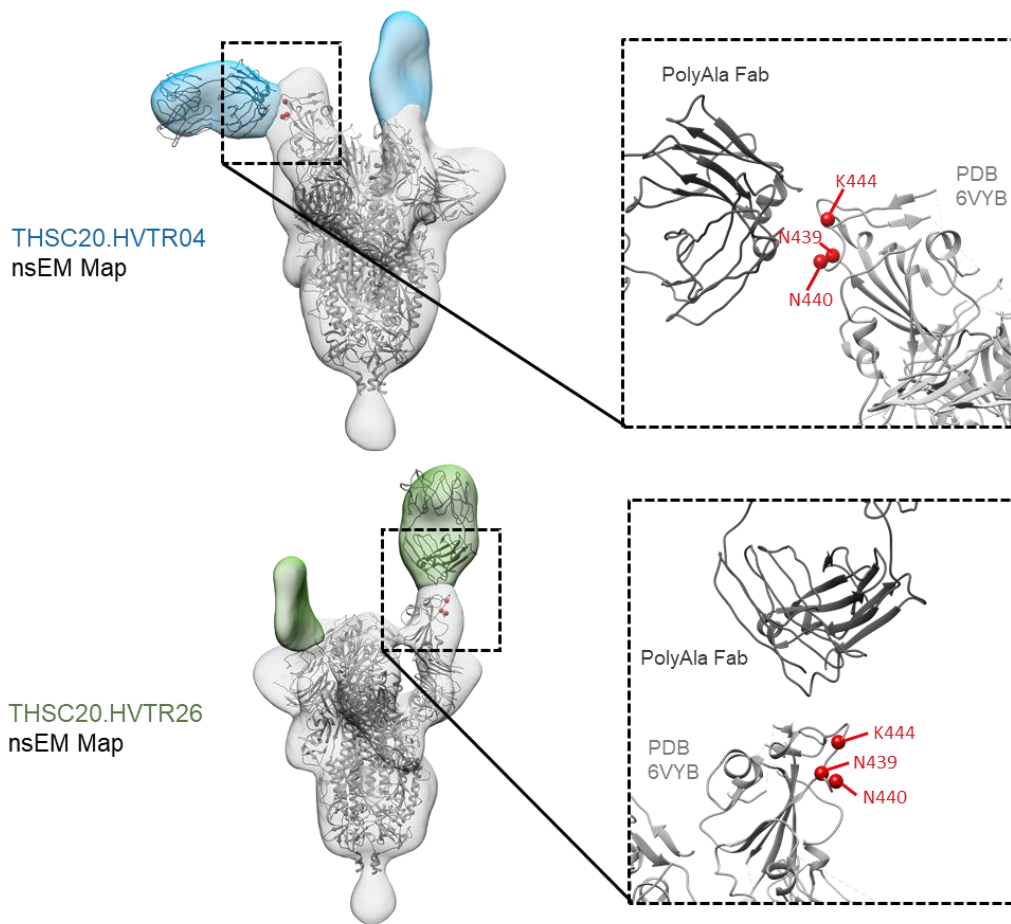

Supplement: S10 Fig — PDB 6VYB and a polyalanine Fab model fit into the spike and fab nsEM densities, respectively. (PDF) [file ppat.1010465.s016.pdf]

## Slide 1
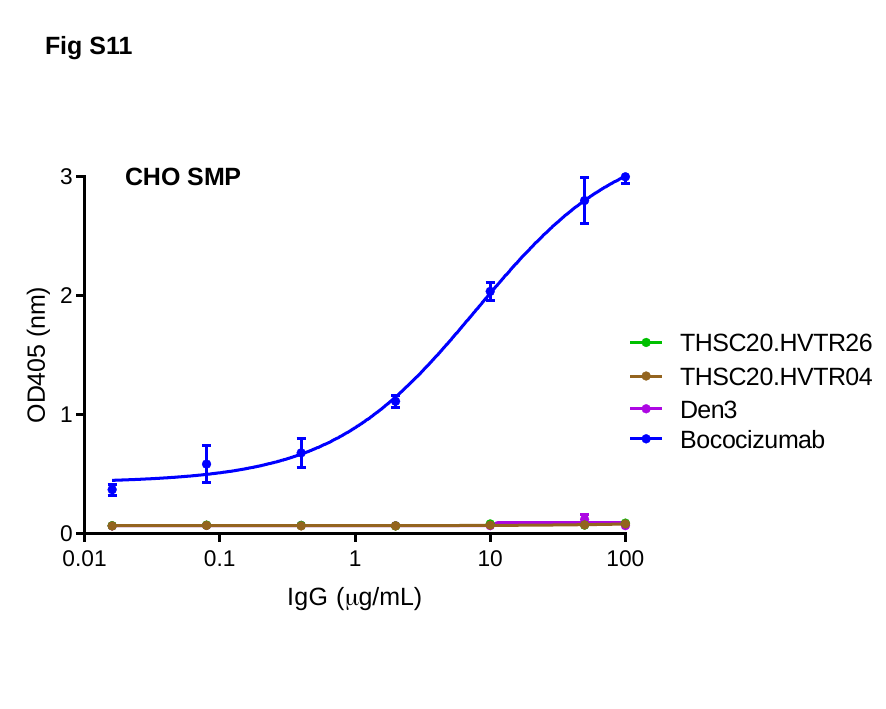

Fig S11

Supplement: S11 Fig — The polyreactivity of THSC20.HVTR04 and THSC20.HVTR26 mAbs using CHO soluble membrane protein (SMP) by ELISA. Three-fold serial dilutions of mAbs starting with 100ug/mL were tested. (PPTX) [file ppat.1010465.s017.pptx]

## Slide 1
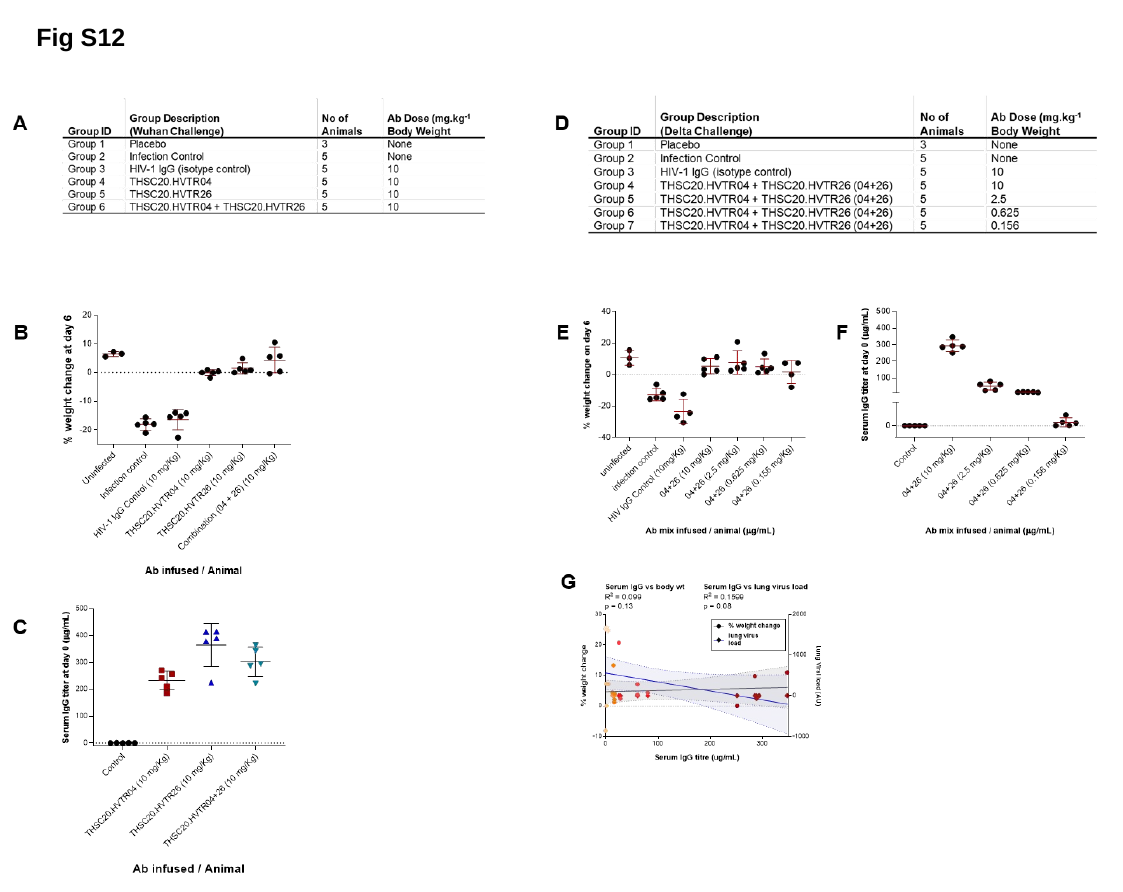

Fig S12

Supplement: S12 Fig — A. Animal grouping and antibody doses given (Wuhan challenge). B. Comparison of body weights between animals that received antibodies and those who did not prior to challenge with Wuhan isolate. C. Quantification of circulating serum IgG in mice at day 0 one day after infusion of mAbs at indicated doses and before virus (Wuhan) challenge. Values represent mean with SEM. D. Animal grouping and different antibody combination doses given (Delta challenge). E. Percent change in body weight of animals that received different doses of mAb combinations. Values represent mean with SEM. F. Quantification of circulating serum IgG concentration in mice at day 0 one day after infusion of mAbs at indicated doses and before virus (Delta) challenge. Values represent mean with SEM. G. Correlation between percent body weight change on day 6, circulating serum IgG concentration on day 0 and lung viral load on day 6 in mice those received different concentrations of mAb combinations. (PPTX) [file ppat.1010465.s018.pptx]
